# Chronic hepatitis in horses with persistent equine hepacivirus infection

**DOI:** 10.1111/evj.70124

**Published:** 2025-12-25

**Authors:** Mason C. Jager, Daniela Luethy, Samantha Shallop, Jessica Cathcart, Thomas J. Divers, Jean‐Yin Tan, Erin McConachie Beasley, Philip Johnson, Laurence Leduc, Claire Smith, Camilla Anne Jamieson, K. Gary Magdesian, Gerlinde R. Van de Walle, Joy E. Tomlinson

**Affiliations:** ^1^ Department of Population Medicine and Diagnostic Sciences Cornell University College of Veterinary Medicine Ithaca New York USA; ^2^ Department of Clinical Studies New Bolton Center, School of Veterinary Medicine, University of Pennsylvania Kennett Square Pennsylvania USA; ^3^ Department of Clinical Sciences Cornell University College of Veterinary Medicine Ithaca New York USA; ^4^ Faculty of Veterinary Medicine and Taylor Institute for Teaching and Learning, University of Calgary Calgary Alberta Canada; ^5^ University of Georgia College of Veterinary Medicine Athens Georgia USA; ^6^ Equine Internal Medicine and Surgery, College of Veterinary Medicine, University of Missouri Columbia Missouri USA; ^7^ Faculté de médecine vétérinaire Université de Montréal Saint Hyacinthe Quebec Canada; ^8^ Sound Equine Veterinary Hospital Pouslbo Washington USA; ^9^ Department of Veterinary Clinical Sciences Purdue University College of Veterinary Medicine West Lafayette Indiana USA; ^10^ Department of Medicine and Epidemiology UC Davis School of Veterinary Medicine Davis California USA; ^11^ Baker Institute for Animal Health, Cornell University College of Veterinary Medicine Ithaca New York USA; ^12^ Present address: Grand Prix Equine Newton Connecticut USA; ^13^ Present address: College of Veterinary Medicine Arkansas State University Jonesboro Arkansas USA; ^14^ Present address: The Royal (Dick) School of Veterinary Studies and the Roslin Institute, University of Edinburgh Edinburgh UK; ^15^ Present address: Department of Clinical Studies New Bolton Center, School of Veterinary Medicine, University of Pennsylvania Kennett Square Pennsylvania USA

**Keywords:** cholangitis, dissecting fibrosis, EMPF, hepatitis C virus, hepatocyte necrosis, horse, liver fibrosis, lymphocytic infiltrate, non‐primate hepacivirus

## Abstract

**Background:**

Equine hepacivirus (EqHV) is closely related to hepatitis C virus (HCV), which causes persistent infection and chronic hepatitis in people. Information on persistent EqHV infection and hepatitis is limited.

**Objectives:**

To report 19 cases of chronic hepatitis and persistent EqHV infection.

**Study Design:**

Mixed retrospective and prospective case series.

**Methods:**

Inclusion criteria were: (1) chronic hepatitis, defined as persistently increased serum liver biomarkers, increased serum liver biomarkers accompanied by histopathological evidence of chronicity, for example, fibrosis, or both; (2) positive serum or liver EqHV RT‐qPCR; and (3) available liver histopathology. Horses were excluded if they became serum EqHV RT‐qPCR undetectable, died, or were euthanised within 6 months of EqHV detection. Liver biopsies were independently reviewed.

**Results:**

Twenty‐nine horses met inclusion criteria. Ten were subsequently excluded (two cleared EqHV, 8 died within 6 months). For the remaining 19 horses, the median duration of documented hepatitis was 18.4 (range, 5.2–120) months and documented EqHV viremia was 14.8 (range, 6.9–55.6) months. Histopathological findings mirrored those seen in humans with chronic HCV including fibrosis, lymphocytic infiltrate, lymphoid aggregates, and individual hepatocyte necrosis. One horse was diagnosed with bacterial cholangiohepatitis, and the remainder had no definitive etiologic diagnosis. Bacterial infection, equine parvovirus‐hepatitis infection, and equine multinodular pulmonary fibrosis were frequent comorbidities.

**Main Limitations:**

A direct causal link between EqHV viremia and hepatitis cannot be made from these data.

**Conclusions:**

Some horses with persistent EqHV infection develop chronic hepatitis and liver failure, with clinical and histopathological findings resembling HCV in humans.

## 1 INTRODUCTION

Equine hepacivirus (EqHV) has been identified as a cause of acute, subclinical, resolving hepatitis in horses,^1^, ^2^, ^3^, ^4^ and is the closest genetic relative of hepatitis C virus (HCV).^5^ In addition to genetic similarity, acute EqHV infection shows clinical similarities to acute HCV infection. In resolving infections, EqHV typically causes mild transient hepatitis coinciding with the time of viral clearance, with mild‐to‐moderate elevations in circulating liver biomarkers, lymphocytic liver infiltrate, and scattered individual hepatocyte necrosis.^2^, ^3^, ^4^, ^6^, ^7^, ^8^

In humans, chronic or persistent HCV infections, defined as infections lasting beyond 6 months, occur in approximately 55%–85% of patients, and are associated with severe chronic hepatitis in about 10%–30% of those cases.^9^, ^10^ In horses, EqHV develops persistent infection longer than 6 months in a potentially lower proportion of infections (0%–57%, *n* = 3–12 horses per study^2^, ^3^, ^7^, ^8^ and 8% of 176 seropositive horses in unpublished data of author JET), except for one study from Mongolia which showed persistence in 90% of viremic horses.^11^ Although the mild hepatitis of acute EqHV infections might have little clinical significance, a chronic form could have a more serious impact on horse health. However, it is unclear whether horses with persistent EqHV infection develop chronic hepatitis, as is seen with HCV. A 16‐year‐old horse with chronic hepatitis and persistent EqHV infection for 15 months has been reported, but no liver biopsy was performed.^12^ In another report, a 6‐year‐old horse with a 15‐month duration of EqHV infection had hepatitis characterized by fibrosis, biliary hyperplasia, and mixed inflammatory infiltrates.^13^ These histopathological findings are similar to those in chronic HCV,^14^ suggesting the possibility of a similar syndrome in horses.

We hypothesised that EqHV can cause chronic hepatitis in a subset of persistently infected horses. A prospective experimental infection trial to test this hypothesis is not feasible for the following reasons: (1) the low rate of persistent infection in horses, (2) the predicted low rate of disease in persistently infected animals, assuming similar rates as seen in HCV‐infected humans, and (3) the predicted long duration of infection required for chronic disease to set in, as it can take decades for HCV to cause severe hepatitis in humans.^10^ Therefore, our objective was to document a series of cases in which chronic hepatitis was found in persistently EqHV‐infected horses. This report is intended to enhance clinical awareness that hepaciviral hepatitis could be a significant clinical concern and to increase testing of clinical cases, which should improve our ability to infer causality and determine the prevalence and incidence of this condition.

## 2 MATERIALS AND METHODS

### 2.1 Case recruitment

A call for cases was published on the ACVIM list‐serv, Equine Vet‐2‐Vet Facebook page, and via word‐of‐mouth. Inclusion criteria were: (1) chronic hepatitis, (2) positive serum or liver EqHV RT‐qPCR, and (3) available liver histopathology. As serum viremia closely mirrors liver viral load,^2^ formalin fixation degrades RNA, and diagnosis of HCV also relies on serum testing without liver PCR,^15^ liver RT‐qPCR was not required for inclusion. Enrolled cases were subsequently excluded if they became serum EqHV RT‐qPCR undetectable, died, or were euthanised within 6 months of the first EqHV detection, preventing determination of an acute resolving EqHV infection versus persistent infection. Cases were enrolled both retrospectively, using existing medical records and diagnostic testing at the attending veterinarian's discretion, and prospectively starting in 2021.

### 2.2 Definitions

Hepatitis was defined as an increase above the reference interval of two or more serum liver biomarkers, including aspartate aminotransferase (AST, with concurrent normal creatine kinase, CK), sorbitol dehydrogenase (SDH), glutamate dehydrogenase (GLDH), gamma glutamyl‐transferase (GGT), direct bilirubin, and bile acids. Chronic hepatitis was defined as at least 1 month's duration of hepatitis, hepatitis with histopathological findings of chronicity, such as fibrosis, or both.

Persistent EqHV infection was defined as an infection lasting longer than 6 months, based on the definition used for HCV infection of people,^10^ and determined by at least two serial serum or liver EqHV RT‐qPCR positive tests at least 6 months apart.

### 2.3 Data and sample collection and analysis

Case records were reviewed for signalment, presenting complaint, diagnostic imaging, laboratory test results, treatments, and progression. For retrospective cases, monitoring bloodwork was submitted by attending clinicians at their discretion, which was typically every 1–2 months.

Starting in 2021, horses were enrolled prospectively, and serum, EDTA whole blood, and citrate whole blood (when available) samples were collected approximately every 2 months for at least 6 months. Samples were submitted for serum biochemical liver profile (AST, SDH, GLDH, GGT, total bilirubin, indirect bilirubin, direct bilirubin, CK, bile acids, and triglycerides), complete blood count, serum amyloid A, iron indices (iron, total iron binding capacity, and % saturation), and plasma fibrinogen. Nasal swabs were collected at least once for EqHV RT‐qPCR to evaluate possible viral shedding. Equine herpesvirus‐5 (EHV‐5) qPCR was performed by the New York State Animal Health Diagnostic Center (AHDC) on paired nasal swabs and whole blood as a proxy diagnostic test for equine multinodular pulmonary fibrosis (EMPF) after multiple EqHV‐positive hepatitis cases were observed to have EMPF as a co‐morbidity.

Descriptive data are presented as median (range). As biochemistry samples were submitted to multiple diagnostic laboratories with moderately differing reference intervals, biochemical data are presented as a percentage of the maximum limit of the reference interval, with raw data in the Supporting Information.

Viral detection for EqHV and equine parvovirus‐hepatitis (EqPV‐H) was performed by (RT)‐qPCR, as previously described, either at the AHDC or in the author's laboratory.^16^ Horses were also screened for EqPV‐H, as this virus causes acute hepatitis with some overlapping histopathological findings.^16^ For horses with suspect positive EqPV‐H qPCR results, infection status was confirmed with serology (luciferase immunoprecipitation system, that is, LIPS, assay performed as previously described).^17^ Horses with EqPV‐H co‐infection were not excluded as this was considered a potentially important co‐morbidity to describe. Case numbers were insufficient for statistical comparison of EqPV‐H infected and uninfected cohorts; however, data from EqPV‐H‐infected horses are denoted in the figures.

### 2.4 Histological analysis

For independent, blinded histological analysis, a convenience sample of liver biopsies from seven control horses was included for comparison. These horses were determined to be healthy by physical examination, complete blood count, serum amyloid A, plasma fibrinogen, and serum liver biochemical panel. Control horses were serum (RT)‐qPCR negative and seronegative (LIPS assays performed as previously described) for both EqHV and EqPV‐H.^17^ Biopsies for these horses were obtained under the same IACUC protocol. Median (range) age was 12 (6–19) years old; 5 geldings and 2 mares; 4 Warmbloods and 3 Thoroughbreds.

Formalin‐fixed paraffin‐embedded (FFPE) liver tissue or scanned images were analysed by a board‐certified pathologist, author MCJ. Slides were mixed with samples from healthy and EqPV‐H infected horses and blinded for analysis. Slides were stained with haematoxylin and eosin (HE), reticulin, and Masson's trichrome (MT), and labelled with CD3 immunohistochemistry at the AHDC, as previously described.^18^ Each sample was scored as previously described^18^ with the additional criteria of lipidosis or vacuolar change. Fibrosis was scored according to the Batts and Ludwig system as: 0, none; 1, enlarged, fibrotic portal tracts; 2, periportal or portal‐portal septa but intact architecture; 3, fibrosis with architectural distortion but no obvious cirrhosis; 4, probable or definite cirrhosis.^19^

CD3‐labelled slides were scanned at 40× using a Roche iScan HT slide scanner available to the authors at the AHDC. The quantification of CD3‐positive cells in each sample was performed using the Positive Cell Detection feature of QuPath, an open‐source slide viewer and analyser,^20^ with results expressed as a percent of total cells calculated automatically from the algorithm output.

### 2.5 In situ hybridisation (ISH)

RNAScope™ 2.5 HD‐Red ISH (Advanced Cell Diagnostics, Inc.) was applied to FFPE liver biopsies using a Ventana automated stainer (Roche Diagnostics). RNAScope™ 2.5 VS Probe sets V‐NP. Hepacivirus‐NS3‐C1 (Cat. 1030439) for the *NS3* gene of EqHV, and Ec‐PPIB (Cat. 462359) for the positive control gene *PPIB* were applied to all cases with sufficient sample remaining. The probe set RNAScope™ 2.5 VS Probe‐V‐EqPV‐H‐VP1 (Cat. 559999) for the *VP1* gene of EqPV‐H^21^ was applied to all cases that were serum EqPV‐H qPCR positive and had sufficient sample remaining. Sample RNA quality was assessed as poor, fair, or good based on PPIB labelling (Figure S1). Samples with poor RNA quality were excluded from EqHV ISH analysis. EqHV was described as present or absent and EqPV‐H ISH was scored from 0 to 5+, as previously described.

### 2.6 Data analysis

Descriptive data of serum biochemical and haematological findings were summarised based on peak measurements over the entire course of monitoring.

Ordinal fibrosis scores and quantitative CD3+ cells on liver histopathology were compared between cases and controls by the non‐parametric Mann–Whitney test. Statistical analysis was performed in GraphPad Prism for macOS Version 10.4.0 (GraphPad Software, www.graphpad.com). Significance was set at *p* < 0.05.

## 3 RESULTS

### 3.1 Case overview

Twenty‐nine cases were enrolled with chronic hepatitis and EqHV infection. Cases originated from 15 states in the USA and 1 province in Canada. Individual case details are presented in Table S1. A total of 10 cases were excluded after longitudinal monitoring, leaving 19 cases with chronic hepatitis and documented persistent EqHV infection.

Of the 10 excluded horses, two horses apparently cleared the infection (at least one serum RT‐qPCR undetectable), and eight horses were euthanised within 6 months after the first EqHV RT‐qPCR positive test. The two horses that cleared EqHV infection were ultimately diagnosed with bacterial cholangiohepatitis based on neutrophilic inflammation on liver histopathology in one and post‐mortem examination in the other. Of the eight horses that were euthanised within 6 months, one was also diagnosed with bacterial cholangiohepatitis on post‐mortem examination, and a definitive cause of liver disease was not identified in any of the other horses. The most common histopathological findings in the livers of the euthanised horses included bridging fibrosis or cirrhosis (7/8) and lymphocytic cholangiohepatitis (6/8). Intriguingly, EHV‐5 with EMPF or early pulmonary fibrosis was identified in 3/5 horses that received a complete necropsy. Full details about these cases are presented in Table S1 and Data S1.

### 3.2 Cases with persistent EqHV infection

Nineteen horses were enrolled with chronic hepatitis and persistent hepaciviral infection lasting longer than 6 months, as documented by serial RT‐qPCR on serum, liver, or both samples, of which eight cases were enrolled prospectively.

#### 3.2.1 Signalment and history

Horses were median 16 (range, 5–24) years old at diagnosis of hepatitis, with 6 mares, 12 geldings, and 1 stallion, and 9 Thoroughbreds, 3 Warmbloods, 2 Quarter Horses, and 5 other light breeds. Median duration of documented hepatitis was 18.4 (5.2–120) months with median duration of documented EqHV viremia of 14.8 (6.9–55.6) months.

Clinical signs at presentation included weight loss (6), fever (2), colic (2), and one case each with lethargy, dermal masses, acute renal failure after bisphosphonate treatment, ventral oedema, urine dribbling, ataxia, lameness, and poor performance. Hepatitis was incidentally detected in two cases during serum biochemical testing for research purposes.

#### 3.2.2 Serum biochemistry and haematology

**FIGURE 1:**
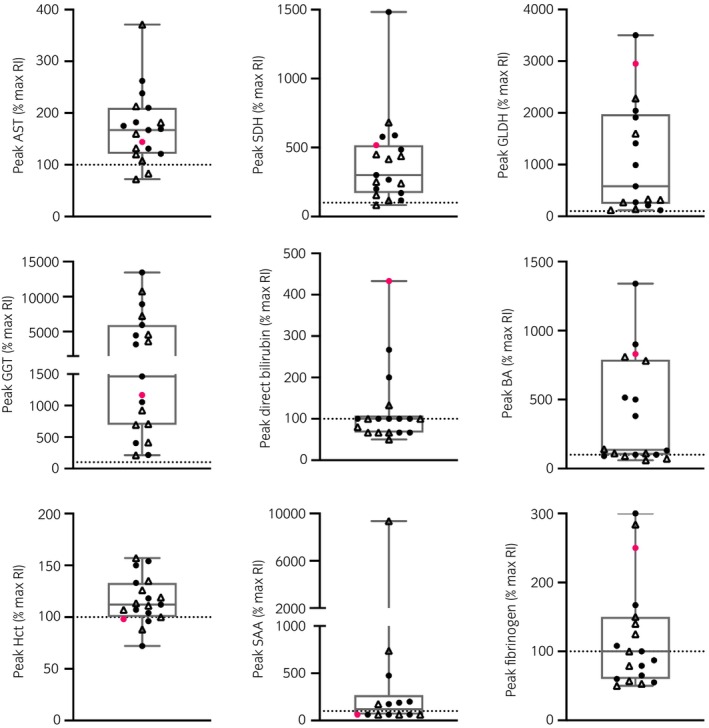
Peak biochemistry, haematocrit and biomarkers of inflammation of 19 horses with chronic hepatitis and persistent equine hepacivirus (EqHV) viremia. Data are presented as percent of maximal end of the reference interval (RI) because samples were tested at various laboratories. Pink, horse with final diagnosis of bacterial cholangiohepatitis; black, horses with no definitive etiologic diagnosis; circles, EqPV‐H negative; triangles, EqPV‐H co‐infected. Dotted line, upper limit of RI.

All 19 horses showed increased GGT and an increase of at least one hepatocellular enzyme activity (AST, SDH, GLDH, or some combination of these; Figure 1, raw data in Figure S2 and Table S2). Peak serum bile acids were also increased in 13/19 horses. Peak total and direct bilirubin were less consistently increased (10/19 and 4/18, respectively). Blood ammonia concentration was measured in only three horses with clinical suspicion of hepatic encephalopathy and was increased in all three horses (Table S2).

Thirteen horses had repeatedly high haematocrit (Hct) in the absence of evidence of haemoconcentration (Figure 1, Figure S2). Leukograms varied with normal, stress leukograms (neutrophilia, lymphopenia), inflammatory leukograms (neutrophilia, monocytosis), mild neutropenia, and reactive lymphocytes observed intermittently across the cases.

Inflammatory markers including serum amyloid A and plasma fibrinogen were evaluated in 14 and 19 cases, respectively. One or both peak inflammatory markers were above the reference interval in 11/19 horses. Minimum serum iron concentration (Fe) and % saturation (Fe sat) were reduced in 1/14 tested cases each. Minimum total iron binding capacity (TIBC) was not reduced in any of the 12 tested cases. In contrast, peak Fe and TIBC were increased in 11/14 and 9/12 horses, respectively, with only 3/12 having increased Fe sat. Concurrently increased Hct (median 50%, range 46%–72%) was observed in 9 of the 11 cases with increased iron indices.

#### 3.2.3 Ultrasonographic examination of liver

**FIGURE 2:**
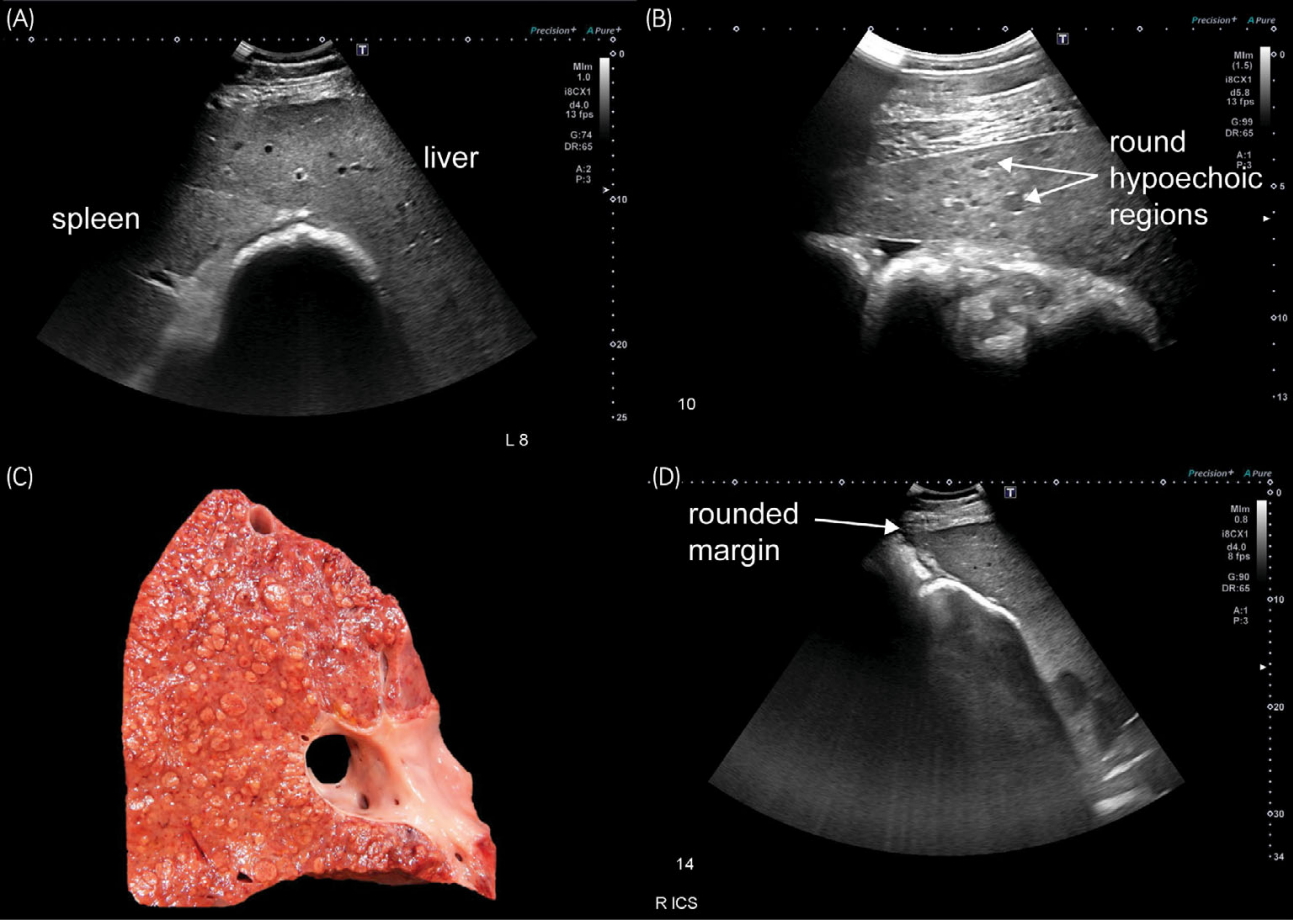
Sonographic findings of horses presenting with chronic hepatitis and equine hepacivirus infection. Findings were variable across horses, and some showed no detected abnormalities. (A) Hyperechoic liver with echogenicity similar to the spleen. Horse RE, EqPV‐H negative. Left cranial abdomen. (B) Heteroechogenic liver with round hypoechoic regions. Horse BE, EqPV‐H negative. Right 10th intercostal space. (C) Sonographic round hypoechoic regions corresponded to cirrhotic nodules. Horse BE, EqPV‐H negative. Post‐mortem right liver lobe cross section photograph. (D) Rounded liver margin. Horse BE, EqPV‐H negative. Right 14th intercostal space.

Sonographic imaging results were available for all 19 cases and findings were highly variable. Alterations in echogenicity were the most common abnormality, with generalised increased echogenicity (5; Figure 2A), heteroechogenicity (4; Figure 2B,C), and scattered hyperechoic foci (4) reported. Hepatomegaly, characterised by subjective enlargement on the left or extension of the liver to the costochondral junction on the right, was observed in five cases and a subjectively small liver was noted in 1 case. In the horse that initially presented with a small liver, the disease progressed over 4 years to hepatomegaly, accompanied by mild ascites, a distended portal vein, and dilated venous architecture. Margins were rounded in five cases (Figure 2D), and distended bile ducts or the parallel channel sign were observed in two cases only. One case had a 1 cm diameter focal shadowing lesion in a bile duct, interpreted as a cholelith. No ultrasonographic liver abnormalities were detected in two cases.

#### 3.2.4 Virology and liver in situ hybridisation (ISH)

**FIGURE 3:**
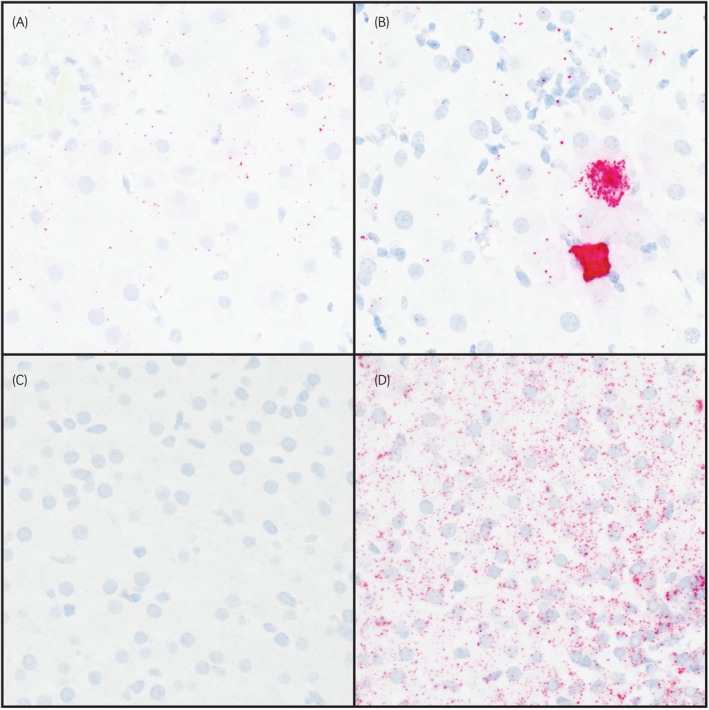
In situ hybridisation (ISH) of equine hepacivirus (EqHV) and equine parvovirus‐hepatitis (EqPV‐H) in horses with persistent EqHV infection. (A) Fine puncta of hybridisation for EqHV nucleic acid were detected in small amounts in hepatocytes throughout lobules, 400X, EqHV ISH. Horse NP, EqPV‐H negative. (B) In cases with both EqHV and EqPV‐H infection, hybridisation for EqPV‐H nucleic acid was often present in large numbers of hepatocytes, 400X, EqPV‐H ISH. Horse DD, EqPV‐H co‐infected. (C) Negative control, no puncta observed in liver tissue from EqHV serum RT‐qPCR negative horse, 400X, EqHV ISH. (D) Positive control, fine puncta of hybridisation for equine housekeeping gene PPIB throughout hepatocytes, 400X, PPIB ISH.

The lowest EqHV Ct (corresponding to the highest viral load) reported per horse was a median of 25 (range, 24–30), corresponding to a median peak viremia of 1.67 × 10^6^ genome equivalents (GE)/mL serum (range, 6.26 × 10^5^–6.67 × 10^7^ GE/mL; Table 1). All horses had serial serum RT‐qPCR testing and the duration of confirmed infection was 14.8 (6.9–55.6) months. All but 3 remained positive at all timepoints tested (see section 3.2.8 Progress and Comorbidities). Eight of the ten cases with fair or high‐quality RNA had positive hybridisation for EqHV in liver tissue, characterised by small numbers of fine puncta in the nucleus and cytoplasm of hepatocytes that were evenly distributed throughout lobules (Figure 3A; Table S1). The two cases that were negative for hybridisation were rated as having fair RNA quality.

**TABLE 1:** Virology results and summary of comorbidities for 19 horses persistently infected with equine hepacivirus.

Horse	Hepatic fibrosis score (0–4)	EqHV viremia (range, GE/mL)	EqHV viremia (range, Ct)	EqPV‐H viremia (range, GE/mL)	EqPV‐H viremia (range, Ct)	EqPV‐H ISH score (0–5+)	EMPF suspicion	PPID	Bacterial infections
AN	4	3.78E4–2.65E6	30.04–34.49	3.4E4–3.1E6^a^	22.05–23.92	5+	Low, EHV‐5 blood (+), nasal swab (−)	No	Yes, cellulitis
DD	3	9.03E5–7.83E6	24.22–28.45	2.8E6–8.8E6^a^	19.04–22.45	4+	Low, terminal increased respiratory rate and effort and cyanosis	Yes	No
JE	2	9.35E4–6.31E5	25.92–28.49	2.3E5–5.2E5^b^	24.03–26.14	3+	No	n/d	No
MA	2	1.86E5–1.80E6	25.15–27.81	1.4E4–2.1E5	27.42–30.98	3+	Moderate, no respiratory signs, blood and nasal swab EHV‐5 (+)	No	No
BR	4	(+), no quantitation	(+), no quantitation	(+), no quantitation	(+), no quantitation	3+	Confirmed by histology, EHV‐5 (+)	Yes	Yes, chronic UTI
TA	1	4.75E4–1.21E6	25.54–30.53	4.3E4–7.9E5^b^	24.52–33.37	n/d	Presumptive asthma, EHV‐5 blood (+), nasal swab (+)	No	Yes, pneumonia, dermatitis, cellulitis
KS	2	ND—2.51E6	27.12–>40	ND–1.87E3	30.04–>40	0	Moderate—respiratory signs with nodular consolidation	No	Yes, pneumonia
BU	2	7.1E4–2.7E6	25.74–29.53	1.7E3–2.3E4	27.88–31.86	0	Moderate—persistent fever, EHV‐5 blood (+), nasal swab (+)	Yes	Presumptive anaplasmosis
AP	n/d	ND—8.96E5	25.91–>40	ND–3.2E4	28.04–>40	n/d	No	No	No
RE	2	ND—4.57E5	23.06–>40	ND	>40	0	No	n/d	Yes, cellulitis
BE	4	(+), no quantitation	25–29.43	ND	>40	0	No	Yes	Yes, cellulitis, sinusitis
TO	4	1.23E4–2.56E6	25.77–37.05	ND	>40	0	No	No	No
OB	3	2.44E4–1.19E6	24.06–31.48	ND	>40	n/d	Moderate, no respiratory signs, EHV‐5 blood (+), nasal swab (+)	No	No
LE	2	1.67E5–6.26E5	24.8–27.62	ND	>40	n/d	No	Yes	No
TE	2	1.92E4–4.53E6	25.58–30.84	ND	>40	n/d	Moderate, increased respiratory rate, B‐lines, EHV‐5 blood (+), nasal swab (+)	No	Yes, multiple hoof abscesses
PA	3	2.83E4–1.59E6	25.34–30.26	ND	>40	n/d	No	No	No
ZG	3	5.13E5–6.58E5	25.64–29.72	ND	>40	n/d	Moderate, no respiratory signs, EHV‐5 blood (+), nasal swab (+)	No	No
HE	2	2.19E5–1.74E6	24.67–28.02	ND	>40	n/d	No	No	No
NP	2	5.85E5–2.36E6	24.75–26.88	ND	>40	n/d	No	No	Yes, cholangio‐hepatitis

Abbreviations: EHV‐5, equine herpesvirus 5; EMPF, equine multinodular pulmonary fibrosis; EqHV, equine hepacivirus; EqPV‐H, equine parvovirus‐hepatitis; GE, genome equivalent; ISH, in situ hybridisation; n/d, not done; ND, not detected; PPID, pars pituitary intermedia dysfunction; UTI, urinary tract infection.

^a^ EqPV‐H viremia >1E6 GE/mL on consecutive samples over at least a 6‐month period.

^b^ EqPV‐H viremia >1E5 and <1E6 GE/mL on consecutive samples over at least a 6‐month period.

Nine of 19 cases were EqPV‐H positive in at least one serum or liver sample (Table 1). The minimum Ct value for positive horses was median 28 (range, 19–39). Parvoviral load remained persistently above 1 × 10^6^ GE/mL serum in two cases and above 1 × 10^5^ GE/mL serum in two other cases for longer than 6 months, which is atypical of previous reports of EqPV‐H infections where usually viremia falls below 1 × 10^5^ GE/mL serum within a median of 2 months of onset of viremia^22^ and viremia over 1 × 10^5^ GE/mL serum is only detected in approximately 5% of samples from chronically EqPV‐H‐infected horses (unpublished data, author JET). Positive hybridisation for EqPV‐H was detected in 5/9 livers from serum PCR‐positive cases tested, all of which maintained serum viremia over 1 × 10^4^ GE/mL on all tested samples and had ISH hybridisation scores of 3+ or greater (Table 1, Figure 3B). No horse cleared parvovirus viremia during the study.

#### 3.2.5 Clinical liver biopsy and histopathology

Transcutaneous needle biopsy was performed in all but 1 horse, which only had post‐mortem histopathology (as this was a teaching and research horse euthanised due to severe degenerative suspensory ligament disease). Four horses had post‐mortem histopathology. Six horses had serial liver histopathology with greater than 6‐month intervals between samples for which the samples were available for review. Aerobic and anaerobic bacterial culture of liver biopsies was performed in 16/19 cases, and no growth was observed in 12/16. Three cases grew suspected contaminants or non‐pathogenic species on enrichment broth including *Escherichia coli* and *Streptococcus bovis*, *Bacillus* spp., and *Pantoea agglomerans*. The sample from one horse had moderate growth of *E. coli* consistent with histological findings of neutrophilic cholangiohepatitis. A liver mineral and heavy metal panel was performed in 4 horses, with variable findings including mild elevations of selenium, copper, mercury, and cadmium at levels not consistent with toxicity (Table S1).

Morphologic diagnoses and interpretive comments from attending diagnostic pathologists varied and were not consistently blinded to virology results. The most common findings were hepatocyte necrosis (13/19), lymphocytic cholangiohepatitis (11/19), and bridging fibrosis (9/19). Some degree of fibrosis was noted in 12/19 cases on HE stain. Neutrophilic cholangiohepatitis was noted in two horses. Interpretive comments indicated differential diagnoses of viral infection (12/19), chronic active hepatitis (6/19), toxic hepatopathy (5/19; three suggested possible pyrrolizidine alkaloid toxicity), ascending bacterial infection (2/19), or some combination of these.

Progression varied in horses with serial biopsies collected greater than 6 months apart (*n* = 6). A reduction in inflammation and fibrosis was noted during serial liver histopathology in one horse that cleared EqHV infection after a period of more than 6 months of hepatitis and viremia (Figure S3). One horse with early disease had similar degrees of moderate lobular hepatitis and interface hepatitis with a stable score of 2/4 septal fibrosis in biopsies, along with stable biochemical abnormalities over a 1.5‐year interval. The remaining 4 horses showed progression of fibrosis over time with intervals of 10 months to 7.5 years. Histopathological progression was independent of changes in liver enzyme activities, which remained stable (2/4), increased and then declined terminally (1/4, 7.5‐year timeframe), or declined terminally (1/4).

#### 3.2.6 Independent review of liver histopathology

**FIGURE 4:**
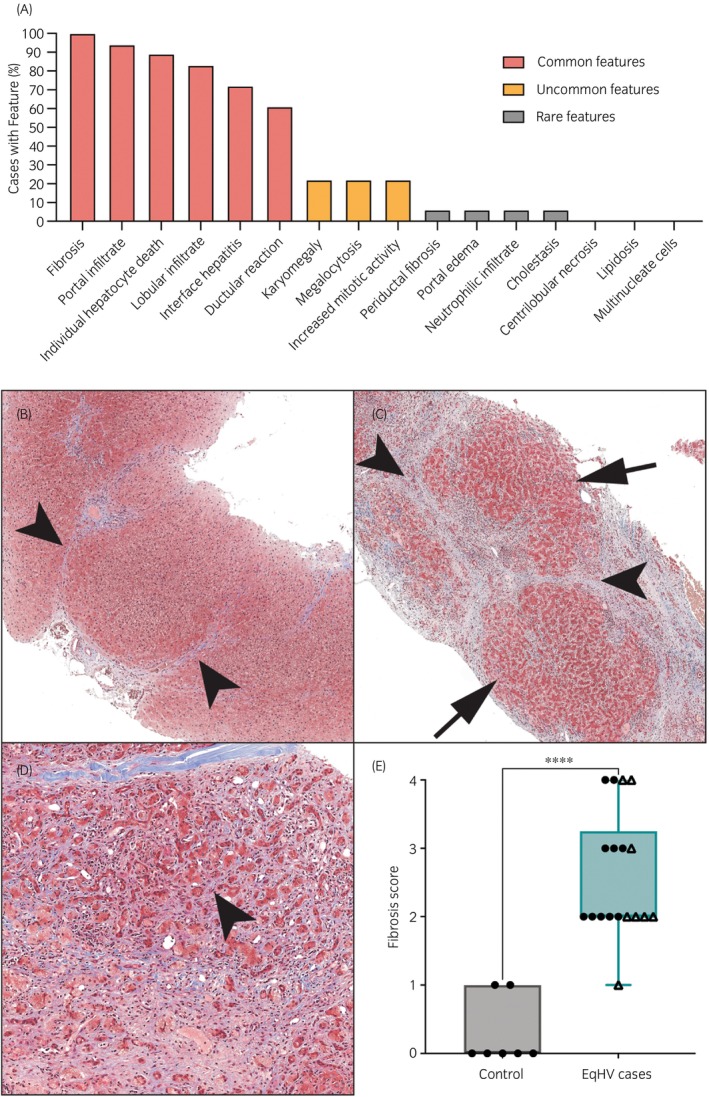
Fibrosis was the most common histological feature identified on blinded independent review of liver biopsies from 18 horses with chronic hepatitis and persistent equine hepacivirus (EqHV) infection. (A) A histogram of the detection rate of each examined histological feature. Each biopsy could demonstrate multiple features. (B) Septal fibrosis with thin septa of collagen that bridge between portal tracts (arrowheads), 50X, Masson's trichrome. Horse RE, EqPV‐H negative. (C) Bridging fibrosis with broad bands of dense collagen (arrowheads) separating nodules of hepatic parenchyma (arrows), 40X, Masson's trichrome. Horse AN, EqPV‐H co‐infected. (D) Sinusoidal fibrosis with thin streams of collagen dissecting between hepatocytes (arrowhead), 100X, Masson's trichrome. Horse BE, EqPV‐H negative. (E) Fibrosis scores were significantly higher in horses with persistent EqHV infection compared to uninfected healthy controls. Mann–Whitney test *p* < 0.0001. Circles, EqPV‐H negative; triangles, EqPV‐H co‐infected.

**FIGURE 5:**
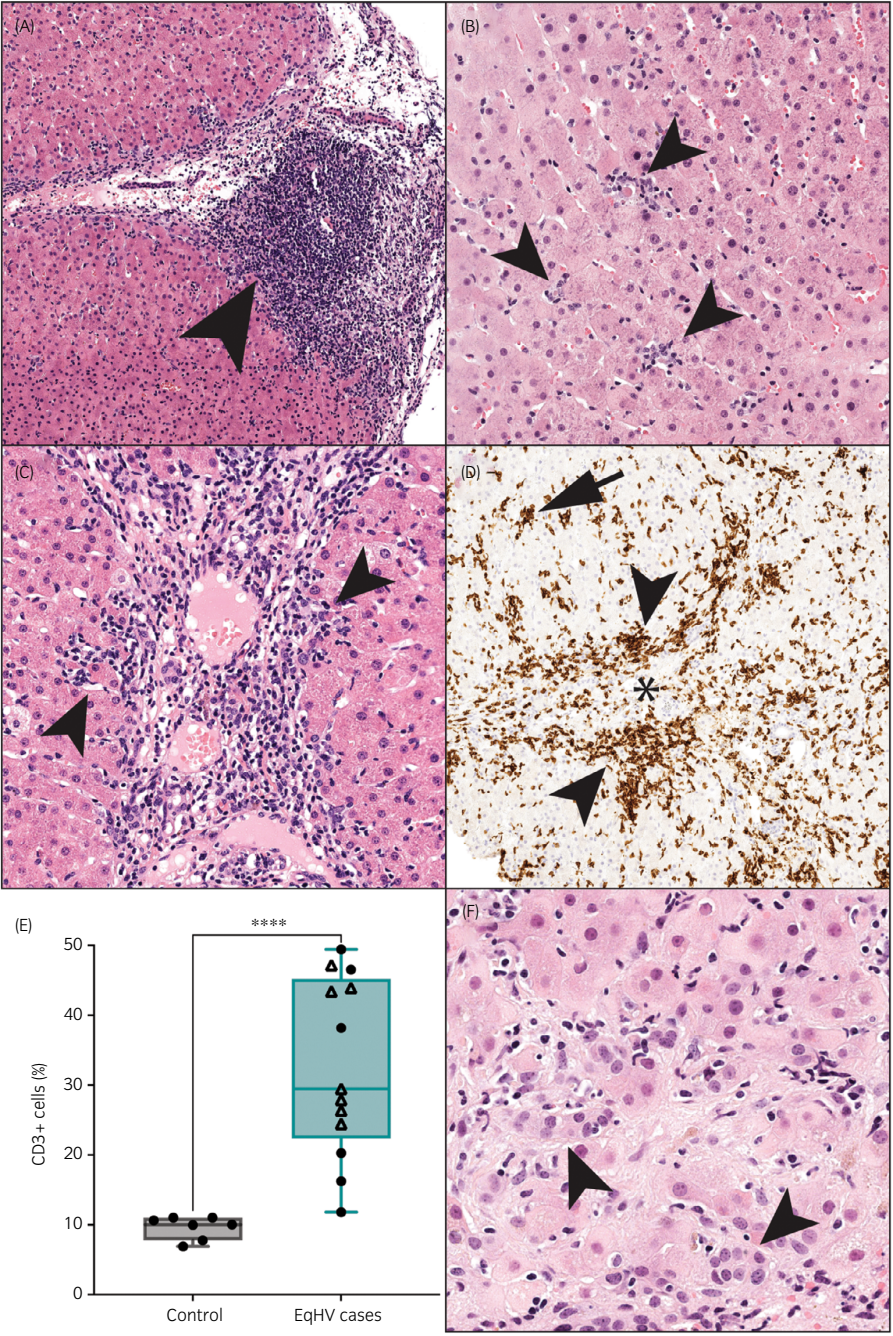
Common histological features of liver biopsies from 18 horses with chronic hepatitis and persistent equine hepacivirus (EqHV) infection. (A) An aggregate of lymphocytes in a portal tract (arrowhead), 100X, HE. Horse TE, EqPV‐H negative. (B) Lobular infiltrates of lymphocytes and macrophages associated with individual dead hepatocytes (arrowheads), 200X, HE. Horse TA, EqPV‐H co‐infected. (C) Interface hepatitis with clusters of lymphocytes that obscure the limiting plate, extend into the parenchyma, and are associated with individual dead hepatocytes (arrowheads), 200X, HE. Horse TE, EqPV‐H negative. (D) CD3‐positive T lymphocytes form tight clusters in hepatic parenchyma (arrow) and along the limiting plate (arrowhead) of portal tracts (asterisk), 100X, CD3 immunohistochemistry. Horse OB, EqPV‐H negative. (F) The percent of CD3‐positive T lymphocytes was significantly higher in horses with persistent EqHV infection compared to uninfected healthy controls (Mann–Whitney test *p* < 0.0001). Circles, EqPV‐H negative; triangles, EqPV‐H co‐infected. (F) Ductular reaction with formation of small, duct‐like structures without lumina, that breach the limiting plate and extend into hepatic cords (arrowheads), 400X, HE. Horse AN, EqPV‐H co‐infected.

Slides or scanned images were available for independent review from 18 out of the 19 cases. Common features detected by blinded evaluation of liver histopathology with combined HE and MT stains included fibrosis, lymphocytic infiltrates, individual hepatocyte death, and ductular reaction (Figures 4 and 5, additional examples Figure S4).

Fibrosis was observed on MT‐stained slides from all horses, despite not being detected based on HE staining assessed by attending pathologists in 7/18. Major patterns included septal (11/18), bridging portal‐to‐portal (5/18), and portal (2/18) fibrosis (note: horses could have more than one pattern, Figure 4B,C). Sinusoidal fibrosis (6/18, Figure 4D) was observed as a minor pattern associated with bridging or septal fibrosis. Semi‐quantitative fibrosis scores were 1–4 out of 4 on blinded independent review of liver histopathology, which was significantly higher than fibrosis scores of control liver biopsies (range 0–1, Mann–Whitney test *p* < 0.001, Figure 4E).

Portal, lobular, and interface inflammation were frequently observed (Figure 5A–C), with some type of inflammation observed in 17/18 cases. Neutrophilic infiltrates were the predominant cell type only in one case, which had a final diagnosis of bacterial cholangiohepatitis. Mononuclear cells predominated in all other cases and immunohistochemistry confirmed the cells expressed CD3, consistent with T‐cells (Figure 5D). Digital image analysis quantitation of CD3+ cell infiltrates showed significant inflammation in cases versus controls (Mann–Whitney test *p* < 0.001, Figure 5E). In some cases, the lymphocytes formed portal aggregates (Figure 5A and Figure S4C).

Individual hepatocyte death was detected in 16/18 cases and was often associated with inflammatory cell infiltrates (Figure 5C). Ductular reaction was observed in 11/18 cases (Figure 5F). Collapse of the reticulin meshwork, consistent with parenchymal loss, was observed in 10 cases.

#### 3.2.7 Treatments

Treatments, determined by the attending clinicians, were highly variable and none of the treatments resolved or clearly improved disease, except for the one horse diagnosed with bacterial cholangiohepatitis, for which liver biomarkers normalised and clinical signs resolved following a course of antibiotic therapy. Four horses received no specific treatment for hepatitis. Fifteen horses received treatment of which 11 received antibiotics as part of their initial treatment for hepatitis (10 trimethoprim sulfadiazine, 2 minocycline, 2 metronidazole, 1 gentamicin). Other products used for treatment included anti‐inflammatory agents (3 prednisolone, 1 firocoxib, 2 flunixin meglumine), vitamin E (3), lactulose (3), pentoxifylline (3), ursodiol (1), and nutraceutical supplements (6 Platinum Performance Liver Support, 1 S‐adenosylmethionine; Table S1).

Two horses were treated with the HCV antiviral combination sofosbuvir/velpatasvir. Sofosbuvir is predicted to inhibit EqHV based on computer modelling.^23^ An uncharacterised imported generic sofosbuvir/velpatasvir product was administered to one horse at an undisclosed dose, without veterinary guidance. During treatment, the owner reported improved clinical signs, and the viremia dropped from average qPCR Ct 26 to Ct 29. After treatment was discontinued due to financial reasons, the viremia rebounded to pre‐treatment levels (Figure S5A). FDA‐approved sofosbuvir/velpatasvir 400 mg/100 mg tablets for human use (Asegua™ Therapeutics) were administered to one horse at 11 mg/kg (550 kg horse, 12 tablets) PO q24h for 75 days extrapolated from human dosing regimens. During treatment, no side effects were noted, the horse's appetite and weight improved, the liver enzyme activities improved, and the viremia dropped approximately 1 log GE/mL during the first month of treatment (Figure S5B). Viremia and liver enzymes began to increase before the end of treatment and rebounded above pre‐treatment levels once the antiviral was discontinued, although the clinical picture remained improved. This horse cleared viremia over a year after discontinuing the antiviral drug treatment.

#### 3.2.8 Progress and co‐morbidities

Co‐morbidities were common, with 15/19 horses being diagnosed with one or more co‐morbidities during the study (Table 1, Table S1). Five of 18 tested horses had resting ACTH concentrations above the seasonal reference interval, an enlarged pituitary gland on necropsy, or both. Eight of 19 horses developed bacterial infections including distal limb cellulitis (4), pneumonia (2), urinary tract infection (2), chronic frontal sinusitis (1), recurrent hoof abscesses (1), and bacterial cholangiohepatitis (1). One additional horse was treated for presumptive anaplasmosis after detection of a fever.

Respiratory disease was common with 6/19 affected horses. Bacterial pneumonia was confirmed in two horses based on results of culture of trans‐tracheal wash fluid and presumptive asthma was diagnosed in one horse by bronchoalveolar lavage cytology after 3 years of intermittent inhaled corticosteroid treatment. Equine multinodular pulmonary fibrosis was confirmed in one horse at necropsy by histopathology and EHV‐5 PCR. Equine multinodular pulmonary fibrosis was suspected by the attending veterinarian in an additional two horses based on respiratory signs, fever, inflammatory profile on CBC, fibrinogen, serum amyloid A, thoracic sonography, or some combination of these. Antimicrobial‐associated colitis developed in two horses treated with antimicrobials for presumptive cholangiohepatitis or anaplasmosis. One horse had recurrent episodes of unilateral uveitis or possible iridocyclitis associated with severe ocular pain and self‐trauma necessitating enucleation. Because of the frequent diagnosis of EMPF in these and the excluded cases (Data S1), paired whole blood and nasal swabs were tested by EHV‐5 qPCR on 13 cases and both samples were positive in 6/13, including the horse diagnosed with asthma, one of the horses with presumptive EMPF (the other was not tested), and the horse with ocular disease. An additional 2/13 tested positive on blood but negative in nasal swab, and 1/13 tested negative on blood but positive in nasal swab.

**FIGURE 6:**
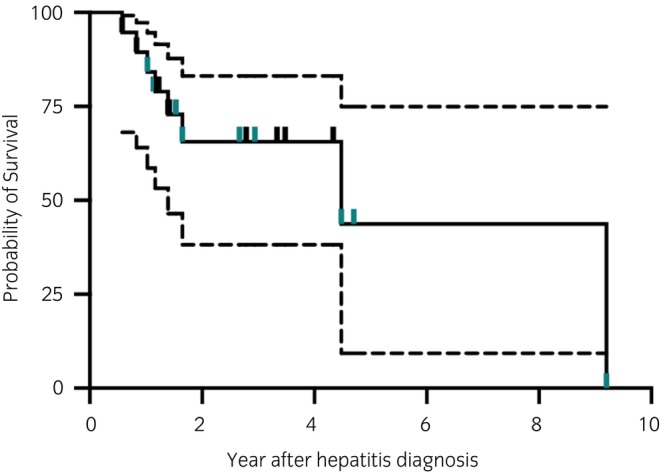
Survival of horses diagnosed with chronic hepatitis and equine hepacivirus infection. Horses were included if there was at least 6 months documented duration of EqHV infection. Tick marks without change in survival probability indicate right censoring (lost to follow‐up), dotted lines indicate 95% confidence interval of survival probability, teal marks reflect data from horses co‐infected with EqPV‐H.

The horse with a confirmed diagnosis of bacterial cholangiohepatitis survived but remained hepacivirus RT‐qPCR positive through the duration of the study. For the remaining 18 cases, 8/18 were euthanised during the study period (Figure 6), with 4 of those for reasons associated with hepatitis. One horse that was diagnosed with hepatitis at 5 years old was euthanised 9 years later (3 years of documented viremia) due solely to progression of hepatitis. The second horse developed progressive weight loss and was euthanised due to acute respiratory distress, cyanosis, and recumbency. No post‐mortem examination was performed. The third horse was euthanised due to clinical decline indicated by progressive weight loss and worsening signs of hepatic encephalopathy (grade 2–3 ataxia, dullness), despite marked reduction in GGT activity (Figure S5C). The fourth horse remained clinically stable for 3 years after diagnosis before showing normalised AST and rising GGT (Figure S5D), inappetence, weight loss, distal limb oedema, and recurring bacterial infections of the skin and lungs. Repeat abdominal ultrasonography revealed the development of ascites and portal vein distention with prominent hepatomegaly. The horse was euthanised due to continued decline in appetite and condition.

Euthanasia or death of the remaining four horses was due to severe suspensory ligament degeneration (1), progressive ulcerative dermal lesions (1), anaphylactic reaction and acute death after ceftiofur crystalline free acid (Excede®, Zoetis Inc.) administered to treat cellulitis (1), and persistent fevers of 104°F and suspected EMPF (1). In the latter horse, biochemical evidence of hepatitis had resolved before euthanasia while remaining serum EqHV RT‐qPCR positive. No necropsy was performed.

Of the 11 horses that survived through the study monitoring, seven had clinically stable liver disease, with stable or decreasing liver enzyme activities (representative case, Figure S5E). One horse showed waxing and waning liver enzymes, with periods of normality between 11.4 and 30.3 months of monitoring. A single undetectable serum EqHV PCR was obtained at 30 months. No follow‐up was available to determine whether the horse remained serum RT‐qPCR negative (Figure S5B). A second horse presumably cleared infection. That horse became serum RT‐qPCR undetectable between samples at 9 and 13 months of monitoring and remained negative on repeated samples. Hepatitis markedly improved after viral clearance based on serum biomarkers and serial liver histopathology (Figures S3 and S5F). A third horse showed slow resolution of hepatitis, followed by significant variability in viremia such that he intermittently tested serum RT‐qPCR negative (Figure S5G).

## 4 DISCUSSION

We have presented 19 horses with persistent EqHV infection >6 months duration and chronic hepatitis and found that cases in this series showed marked diagnostic similarities to Hepatitis C in humans. Histopathological features of viral hepatitis in humans include portal and lobular mononuclear cell inflammation, fibrosis, necrosis, ductular reaction, and lipidosis.^24^ These abnormalities can progress to cirrhosis and hepatocellular carcinoma over time.^14^ Except for lipidosis and carcinoma, all the above‐listed features were observed frequently in the horses included in this report. Not all liver biopsies of every infected horse showed every single histopathological feature, which is similar to the situation reported for human livers infected with HCV.^24^ Portal tract lymphoid aggregates or clusters were observed in some of the horses, and while not pathognomonic, they are highly characteristic of HCV in humans.^24^ Unfortunately, these constellations of histopathological findings are not specific enough to definitively indicate a viral cause, much less specifically a hepaciviral cause.

The diagnostic approach in this study involved ruling out other causes of equine hepatitis. Although initial biopsy reports for 68% of the persistently infected horses mentioned lymphocytic or neutrophilic cholangiohepatitis, only one had a definitive diagnosis of bacterial cholangiohepatitis based on bacterial culture of liver biopsies and response to treatment with antimicrobial therapy. An additional 3 cases in the group of 10 excluded horses were diagnosed with bacterial cholangiohepatitis. Two of those had resolving hepaciviral infection, highlighting the diagnostic challenge of assessing the role of hepacivirus infection based on a single timepoint serum RT‐qPCR. Clinical features typical of bacterial infection, including fevers and increased inflammatory biomarkers, did not reliably separate these four horses with bacterial cholangiohepatitis from the other horses in the study, as co‐morbidities known to cause fevers and inflammation were common. Ascending bacterial cholangiohepatitis is important to diagnose as it is frequently treatable.^25^ The most important diagnostic criteria to differentiate bacterial cholangiohepatitis/cholelithiasis from the other cases were ultrasonographic findings of distended bile ducts or choleliths, liver biopsy with positive culture, and histopathology showing neutrophilic inflammation and ductular reaction, although not all these findings were present in every case. These cases highlight the fact that detection of hepacivirus infection in a horse with hepatitis does not necessarily imply the virus is the primary cause of disease.

Unfortunately, rigorous investigation of all possible alternative causes, particularly regarding toxins, was not consistently performed in all cases. In a single case, acute exacerbation of hepatic enzyme activities coincided with observation of *Cymadothea trifolii* growth on white clover in the pasture; however, hepatitis was also observed at times when the horse was not exposed to that potentially toxic fungus. High copper content was reported in one horse, although the significance of the finding is unknown as this can be observed in many species as a consequence of chronic inflammation and cholestasis rather than a cause of disease.^26^ No suspected source of toxins was identified in any other case, and the majority of horses in this series did not live in the geographic range of plants containing pyrrolizidine alkaloid toxins (a toxic cause of fibrosing hepatitis).^27^ Clinical cases of aflatoxicosis in horses have rarely been reported, and histopathological signs consist predominantly of centrilobular necrosis and lipidosis, which were not observed in these cases.^28^

After ruling out other potential causes of hepatitis, there remain major difficulties in determining whether liver disease in individual horses is caused by hepaciviral infection. First, in severe progressive cases, it is currently impossible to determine whether hepaciviral infection is persistent because horses succumb to disease before the 6‐month monitoring period is complete. Second, since most hepatocytes are infected (as indicated by ours and others' ISH findings)^29^ and pathology is diffuse and chronic, we cannot use histological studies (such as immunohistochemistry or ISH) to determine whether hepacivirus is directly tied to the observed pathology. That approach has been successfully employed to demonstrate that, in the context of EqPV‐H, necrotic cells are infected and surrounded by inflammatory cells.^22^ These limitations, combined with the inherent limitations of a descriptive case series, indicate that hepaciviral infection could have been incidental in some or all cases presented here.

Although we cannot fulfill the classic version of Koch's postulates with this case series, there is strong evidence supporting the conclusion that EqHV likely causes chronic hepatitis in a subset of horses with persistent infection. In 1996, Fredricks and Relman proposed a new series of criteria for assessing infectious agents that have been identified by sequence analysis,^30^ as is the case for HCV and EqHV. Combining ours and others' published EqHV research with the additional findings reported in this case series, allows for the fulfillment of the majority of these criteria. EqHV is hepatotropic, as demonstrated by ISH and RT‐qPCR, and the highest copy numbers are detected in the liver, which is consistent with liver pathology.^3^, ^4^, ^29^ Experimental infection studies of acute EqHV have demonstrated that sequence detection predates disease and that clinical resolution follows viral clearance.^2^, ^7^, ^8^ Additionally, two horses in this study showed clinical resolution of chronic disease coinciding with viral clearance. This finding should be followed up with longitudinal studies to demonstrate that persistent EqHV infection predates chronic hepatitis. Perhaps most importantly, the nature of the presumed EqHV pathology is consistent with the known biological characteristics of its close genetic relative, HCV.

The cases included in this study present a range of severity and outcomes, with approximately a third of horses dying before infection persistence could be determined. If horses survived the first 6 months after diagnosis and remained persistently EqHV viremic, prognosis was fair for at least 18‐month survival. As has been described for chronic liver disease, increased GGT activity was the most consistent finding, and hepatocellular enzyme activities might be normal or nearly normal. In multiple cases, horses with severely increased GGT activity would show a decline in GGT toward normal over time. It is unclear whether this represented improvement or progression of disease, as most clients declined repeated liver biopsies in cases that showed falling liver enzyme activities without overt progression of clinical signs. Overall, case progression was consistent with prior reports that GGT activities were not accurate prognostic indicators, as horses could remain with GGT >1000 U/L for months or years without obvious clinical signs and conversely, histological damage, fibrosis, and clinical signs progressed in the face of stable or declining liver enzyme activities in some cases in this series.^25^, ^31^

Comorbidities were common in this cohort, likely simply reflecting the long period of monitoring; however four specific comorbidities predominated. First, bacterial infections were frequent. As the liver has primary immunologic functions in reducing the systemic spread of bacteria that translocate from the gastrointestinal system, it is plausible that horses with reduced hepatic function might be more prone to bacteremia and secondary bacterial infections seeding distant locations in the body. An increased risk of bacterial infections has been reported in patients with HCV and cirrhosis of any cause, with commonly observed sites of infection in humans including bacteremia, pneumonia, urinary tract infection, soft tissue infection, and peritonitis,^32^, ^33^, ^34^, ^35^, ^36^, ^37^ most of which were also observed in these equine cases. Second, for being a rather rare disease, a high proportion of enrolled cases (4/29, 14%) were definitively diagnosed with EMPF. Paired EHV‐5 PCR positive whole blood and nasal swabs have been suggested to be indicative of EMPF, with a sensitivity of 90% and a specificity of 89.8%.^38^ An additional six horses with persistent EqHV infection met this diagnostic criterion, four of which had clinical or sonographic evidence of pulmonary disease. The relationship between the two viral conditions is unclear. The prevalence of exposure to both viruses is high, while clinical disease prevalence is low.^38^, ^39^ It is intriguing that the associated diseases both involve fibrotic pathology, suggesting either a possible underlying immunologic bias toward pro‐fibrotic pathways in these horses, or that a pro‐fibrotic response to one infection might promote disease progression for the other virus. Third, 28% of horses were also diagnosed with PPID, which can cause immunosuppression. It is unclear if the high proportion of affected horses is solely due to the age of affected animals, or if PPID contributes to the development of persistent infection or disease. Fourth, concurrent EqPV‐H infection was detected in approximately half the cases and was actively contributing to liver damage in approximately a quarter of cases. This is in comparison to the reported 15% prevalence of EqPV‐H viremia in apparently healthy horses in the United States.^40^ Concurrent EqPV‐H infection might indicate shared transmission pathways such as haematogenous spread via biting flies. Persistent high viral load with EqPV‐H in persistent EqHV infected horses might reflect inherent deficits in antiviral immunity, or EqHV infection might set up a microenvironment that is more permissive to EqPV‐H replication. In some cases, EqPV‐H appeared to be actively contributing to disease based on ISH; however EqPV‐H infection has not been associated with fibrotic liver disease in other studies.^18^ Overall, further information is needed to determine the cause of the apparent associations between EqHV, hepatitis, and these co‐morbidities and to investigate whether they influence case progression and prognosis.

In conclusion, excluding 4/29 cases with a confirmed diagnosis of bacterial cholangiohepatitis, we present 25 cases of possible EqHV‐associated chronic hepatitis, of which 18 met the criteria of persistent EqHV infection. Further epidemiologic and longitudinal studies are warranted to examine the hypothesis that EqHV is a cause of equine chronic hepatitis. Still, there is enough evidence currently to consider that EqHV is likely to be a cause of chronic hepatitis, especially when fibrosis, hepatocyte necrosis, portal lymphoid aggregates, ductular reaction, cholangitis, or a combination of these are observed on liver histopathology. Pathologists are encouraged to use MT stain to observe mild fibrosis which might be overlooked on HE stain. Clinicians are cautioned that a single timepoint EqHV positive RT‐qPCR test outcome cannot be used alone to diagnose EqHV as the cause of hepatitis in an individual patient and that co‐morbidities including EqPV‐H and bacterial infections of the liver must be considered.

## FUNDING INFORMATION

National Institutes of Health, National Institute of Allergy and Infectious Diseases K08AI141767; National Institutes of Health, National Institute of Allergy and Infectious Diseases K08AI63401; National Institutes of Health, NIH Office of the Director T32ODO011000; U.S. Department of Agriculture, National Institute of Food and Agriculture 2022‐67015‐36343.

## CONFLICT OF INTEREST STATEMENT

The authors declare no conflicts of interest.

## AUTHOR CONTRIBUTIONS

**Mason C. Jager:** Conceptualization; investigation; writing – original draft; methodology; validation; visualization; writing – review and editing; formal analysis. **Daniela Luethy:** Conceptualization; writing – review and editing. **Samantha Shallop:** Investigation; methodology; writing – review and editing. **Jessica Cathcart:** Data curation; writing – review and editing. **Thomas J. Divers:** Conceptualization; investigation; project administration; writing – review and editing. **Jean‐Yin Tan:** Writing – review and editing; investigation. **Erin McConachie Beasley:** Investigation; writing – review and editing. **Philip Johnson:** Investigation; writing – review and editing. **Laurence Leduc:** Investigation; writing – review and editing. **Claire Smith:** Investigation; writing – review and editing. **Camilla Anne Jamieson:** Investigation; writing – review and editing. **K. Gary Magdesian:** Investigation; writing – review and editing. **Gerlinde R. Van de Walle:** Investigation; conceptualization; funding acquisition; writing – review and editing; resources. **Joy E. Tomlinson:** Conceptualization; investigation; funding acquisition; writing – original draft; methodology; validation; visualization; writing – review and editing; software; formal analysis; project administration; data curation; supervision; resources.

## DATA INTEGRITY STATEMENT

Joy E. Tomlinson had full access to all the data in the study and takes responsibility for the integrity of the data and the accuracy of the data analysis.

## ETHICAL ANIMAL RESEARCH

Prospective samples were collected under IACUC #2014‐0024 from Cornell University.

## INFORMED CONSENT

Informed consent was obtained.

## Supporting information


**Data S1.** Summary case information for the 10 cases excluded due to viral clearance or death within 6 months of initial EqHV detection.

**Figure S1.** Examples of RNA integrity assessment in formalin‐fixed paraffin embedded equine liver samples using in situ hybridisation of the equine *PPIB* housekeeping gene. Expression should be ubiquitous in equine hepatocytes. (A) Poor RNA quality with <10 puncta per cell (B) Fair RNA quality with 10–20 puncta per cell and (C) Good RNA quality with >21 puncta per cell, 400X, PPIB ISH. Samples with poor quality labelling with PPIB were not evaluated by EqHV ISH.

**Figure S2.** Peak biochemistry and haematology of all 29 horses presented with chronic hepatitis and equine hepacivirus viremia. Samples were tested at various laboratories and reference intervals varied. Horses were grouped as Acute (cleared viremia within 6 months), <6 mo. (died or euthanised with <6 months documented viremia), and >6 mo. (documented persistent viremia >6 mo. duration, included in main manuscript). Pink squares, horses with final diagnosis of bacterial cholangiohepatitis; teal circles, values within lab‐specific reference intervals; black circles, values above lab‐specific reference intervals.

**Figure S3.** Progression of disease after viral clearance, case RE. (A) Liver biopsy from week 0 with lymphocytic and neutrophilic portal infiltrates, early bridging fibrosis, lobular infiltrates with scattered individual hepatocellular necrosis (arrow), and ductular reaction (arrowhead). Masson's trichrome stain was not performed on this sample. (B) Liver biopsy from week 51, shortly after viral clearance, with reduced portal and lobular inflammation (arrowhead). (C) Liver biopsy from week 51 with persistent fine septa of collagen between portal tracts (arrowhead) and between portal tracts and central veins (arrow). (D) Liver biopsy from week 65 with only occasional fine septa between portal tracts (arrowhead). Severity of fibrosis and inflammation are reduced compared to week 51. A and B HE. C and D Masson's trichrome. Scale bars: A = 50 μm, B = 100 μm, C and D = 200 μm.

**Figure S4:** Additional examples of histopathological features of cases with documented persistent hepacivirus infection. (A) Dissecting fibrosis (arrow) separating and isolating islands of hepatocytes (arrowhead), case BE. (B) Septal and dissecting sinusoidal fibrosis (arrow), case ZG. (C) Dense lymphoid aggregate (arrowhead) expanding a portal tract, case BE. (D) Clustered necrotic hepatocytes (arrow) and adjacent macrophages and lymphocytes (arrowhead), case BE. (E) Collapse of the reticulin meshwork and condensation of fibres in sinusoids (arrowhead), case BE. (F) Mild ductular reaction indicated by cords of progenitor cells that breach the limiting plate and transition into hepatic cords (arrowheads), case RE. A and B Masson's trichrome. C, D, and F HE. E reticulin. Scale bars: A–C, E = 100 μm; A inset = 800 μm; B inset = 200 μm; D = 20 μm; F = 50 μm.

**Figure S5:** Timeline of liver biomarkers and serum viremia for horses with hepaciviral infection and chronic hepatitis. (A) Horse BE, 16‐year‐old Anglo‐Arabian gelding. Viremia declined during treatment with antivirals, but rebounded after treatment. Died from anaphylactic drug reaction. (B) Horse KS, 16‐year‐old TB stallion. Viremia and liver enzymes declined during treatment with antivirals, but rebounded during and after treatment. EqHV later dropped below the limit of detection and resolved hepatitis after >6 mo persistent infection. (C) Horse AN, 15‐year‐old Thoroughbred gelding with markedly increased GGT activity (>2000 U/L) that began to decline over time (to 600 U/L) despite increased severity of clinical signs. Euthanised. (D) Horse TA, 10‐year‐old Thoroughbred mare. AST normalised but GGT increased concurrent with progression of clinical signs and sonographic evidence of portal hypertension. Euthanised. (E) Horse TE, 6‐year‐old Thoroughbred gelding. Apparently stable liver enzymes, viremia, and histology over 2 years. (F) Horse RE, 12‐year‐old Thoroughbred gelding spontaneously cleared EqHV and resolved hepatitis after >6 months persistent infection. (G) Horse AP, 18‐year‐old appendix Quarter Horse gelding with resolved hepatitis after >6 months persistent infection, followed by variable viremia that fell below the limit of detection at one timepoint.

**Figure S6.** Sonographic findings in horses with bacterial cholangiohepatitis and equine hepacivirus infection. (A) Hyperechoic liver with biliary distention and multiple hyperechoic, variably shadowing foci consistent with choleliths in a horse with confirmed bacterial cholangiohepatitis. Horse QL. Left 7th intercostal space. (B) Parallel channel sign indicating distended bile ducts. Horse OW. Right abdomen.

**Figure S7:** Histopathologic features of cases WE and OW. (A) Severe portal to portal bridging fibrosis.

**Table S1.** Individual case information for all cases enrolled, including those later excluded due to viral clearance or death within 6 months of initial EqHV detection. Cases are organised by presence or absence of EqPV‐H co‐infection.

**Table S2.** Peak biochemical markers and complete blood count findings for horses enrolled with chronic hepatitis and equine hepacivirus viremia. Horses were grouped as Acute (cleared viremia within 6 months), <6 mo. (died or euthanised with <6 months documented viremia), and >6 mo. (documented persistent viremia >6 mo. duration, included in main manuscript). Data presented as median (range).

## Data Availability

The data that support the findings of this study are openly available in ScholarlyCommons at http://doi.org/10.48659/7nfx-7731, reference number 20.500.14332/62219.
