# Supplementary material for: Chronic hepatitis in horses with persistent equine hepacivirus infection
Source: Equine Vet J. 2025 Dec 25;58(2):444–57. doi: 10.1111/evj.70124 (PMC12892389; doi:10.1111/evj.70124)
Supplement: Supplementary file 1 — Data S1. Summary case information for the 10 cases excluded due to viral clearance or death within 6 months of initial EqHV detection. [file EVJ-58-444-s003.pdf]

Data S1: Enrolled and later excluded cases.

1. Cases with EqHV infection resolving within 6 months of initial detection

Two horses presented with chronic hepatitis and EqHV infection but cleared the infection at 3 weeks and 3 months, respectively, of initial viral detection (**Tables S1, S2, Figure S2**). Horse QL, a 7-year-old Thoroughbred gelding, presented with recurrent colic, fevers, liver enzyme elevations, elevated inflammatory biomarkers including plasma fibrinogen and serum amyloid A (SAA), and sonographic evidence of hepatomegaly, multifocal hyperechoic foci with variable shadowing, biliary distention (parallel channel signs), increased bile echogenicity, and liver hyperechogenicity **(Figure S6A)**. After 2 months with poor response to antimicrobial treatment, the horse was euthanised and bacterial cholangiohepatitis was confirmed by liver culture and histopathology. Horse HU, a 10-year-old Warmblood mare, presented with increased liver enzyme activities that developed during treatment for rhabdomyolysis, colic, post-celiotomy incisional infection, and equine squamous gastric ulcer disease. The horse also had elevated inflammatory biomarkers at the time the increased liver enzymes were first detected but no significant sonographic findings in the liver. For the multiple comorbidities, the horse was treated with antimicrobials, misoprostol, levothyroxine, altrenogest, and various supplements including milk thistle (Equine Liver Flush®, Omega Alpha Equine USA, Ontario, CA) over a 5-month period. Although histopathology was consistent with bacterial cholangiohepatitis with findings of mild, chronic, neutrophilic and histiocytic lobular hepatitis with mild ductular reaction, the prescribed treatment approach was to discontinue all medications and supplements. Subsequently, EqHV infection cleared (serum RT-qPCR undetectable) and hepatitis resolved.

2. Cases euthanised within 6 months

Eight horses had severe liver disease at initial diagnosis and were euthanised within 6 months after first EqHV RT-qPCR positive test (**Table S1)**. A definitive cause of liver disease was not identified in 7 horses. One 4-year-old Thoroughbred gelding presented with multiple limb lameness and intermittent fever and was clinically diagnosed with hepatitis with sonographic evidence of biliary distention (parallel channel sign) but no growth on culture of liver biopsy. The horse was euthanised 5 weeks after first presentation

and was diagnosed with bacterial cholangiohepatitis and cholelithiasis on post-mortem examination (Horse OW, **Table S1**). All 8 cases are presented below.

3.2.1 Signalment and presenting complaint:

Horses were 15 (range, 4-19) years old, with 7 geldings and 1 mare. There were 6 Thoroughbreds and 2 Warmbloods. Follow-up information was available for median documented duration of hepatitis of 2.8 (0.5 – 5.6) months, and EqHV infection of 1.3 (0.3 – 4.5) months.

Clinical signs at presentation included weight loss (4), inappetence (4), lethargy (3), increased rectal temperature (5), cough (1), lameness (1), and poor performance (1).

3.2.2 Serum biochemistry and hematology:

Liver markers were moderately to markedly increased with mixed hepatocellular leakage and induction enzymes involved (**Figure S2, Table S2**) and normal CK. Serum bile acid concentrations were also increased in 7/7 tested horses. Total and direct bilirubin concentrations were increased in 6/8 and 2/8 horses, respectively. Blood ammonia concentration was measured in 2 horses and was increased in one with signs of altered mentation and ataxia (186 µg/dL; RI 13- 108 µg/dL; horse GA).

Three of 8 cases had repeatably high hematocrits (Hct, 48 – 61%; RI, 32 - 47%) and red blood cell counts (12.2 – 13.3 million/µL; RI, 6.6 – 9.7 million/µL), in the absence of clinical evidence of hemoconcentration (**Figure S2, Table S2**). Normal leukograms (n = 4) or neutrophilia (n = 4, peak 9.2 – 22.3 $\times 10^3$ neutrophils/µl; RI, 2.7 – 7.0 $\times 10^3$/µl) were predominantly observed. Peak plasma fibrinogen concentration was above reference interval in 3/7 horses with median 300 mg/dL (range 104 – 900 mg/dL; median 71%, range 46 – 300% of RI). Serum amyloid A (SAA) concentration was measured in 3 horses and was increased in 2/3. Serum iron concentration (Fe) and total iron binding capacity (TIBC) are reduced in inflammatory states. These indices were measured in two horses and were normal in one (horse LA) with a normal Hct and increased in the second (horse GA) with an increased Hct of 61%.

3.2.3 EqHV detection:

Viral load varied with median minimum serum Ct 23.89 (range, 20.7 – 30.7; n = 8) and fresh liver Ct 29.1 – 39.0 (n = 4). Serum was not available for quantitation, except in one horse with 4.57 $x10^5$ genome equivalents (GE)/ml at a single timepoint.

3.2.4 Ultrasonography:

Sonographic findings included altered liver size (5/8, 63%), echogenicity (3/8, 38%), rounded margins (2/8, 25%), and distended bile ducts (2/8, 25%). Size was reported as increased in 4, reduced in 1, and normal in 3 horses. Echogenicity was diffusely increased in 1, heteroechoic in 1, and characterised by multiple hyperechoic foci with inconsistent shadowing in 1. The hyperechoic foci were determined to be micro-abscesses and not choleliths on post-mortem examination. Margins were mildly rounded in 1 horse and irregular with nodular appearance in 1 horse. Occasional parallel channel sign and distended bile ducts with sludge and shadowing choleliths were observed in the horse with bacterial cholangiohepatitis (**Figure S6B,** horse OW).

3.2.5 Liver biopsy and histopathology:

Transcutaneous needle biopsy was performed in 7/8 and post-mortem histopathology was performed in all 8 horses. Bacterial culture was performed in all cases and no growth (3) or light growth of suspected contaminants (4; 2 *Staphylococcus aureus*, 1 *Streptococcus bovis*, 1 *Acinetobacter spp.*) were observed. On the initial biopsy the attending pathologist's interpretations included bridging fibrosis or cirrhosis (7/8) and lymphocytic cholangiohepatitis (6/8) as the most common findings. Necropsy findings were largely similar to ante-mortem biopsy findings, except for horse OW, for which no etiology was identified on the ante-mortem biopsy and bacterial cholangiohepatitis was diagnosed on the necropsy sample.

Necropsy samples were available from 7/8 horses for independent blinded review (**Figure S7**). The necropsy sample from the eighth horse showed freeze artifact preventing evaluation of cellular morphology but not evaluation of collagen on Masson's trichrome. Fibrosis was observed in all 8 cases with scores of 3 – 4 out of 4. Portal infiltrate, interface inflammation, and ductular reaction were observed in all 7 cases. Other findings included individual hepatocyte necrosis (6/7, 86%), lobular infiltrate (5/7, 71%), karyomegaly

(4/7, 57%), megalocytosis (2/7, 29%), cholestasis (2/7, 29%), neutrophilic infiltrate (1/7, 14%), and multinucleate hepatocytes (1/7, 14%). Increased mitotic activity, periductal fibrosis, portal oedema, centrilobular necrosis, and lipidosis were not observed.

3.2.6 Treatments:

Treatments were highly variable and included antibiotics in 6, anti-inflammatory agents (corticosteroids in 5, flunixin in 3, pentoxifylline in 4), and nutraceutical supplements including vitamin E, silybin (a.k.a. milk thistle), and S-adenosylmethionine (SAMe) in 5 (**Table S1)**. None of the treatments were temporally associated with resolution or clear improvement in disease.

3.2.7 Progress and co-morbidities:

Co-morbidities were common. Five horses (6/7, 86%, one horse was not tested) were serum and/or liver EqPV-H qPCR positive. Three showed high parvoviral load with minimum Ct 18.32 – 21.34 consistent with active parvoviral disease contributing to the overall pathology**.** Equine herpesvirus-5 (EHV-5) with equine multinodular pulmonary fibrosis (EMPF) or early pulmonary fibrosis was identified in 3/5 horses with complete necropsy (60%). Bacterial co-infections were also common and were observed in 3/7 (43%) with 1 case each of *R. equi* pulmonary abscess, pleuropneumonia, and distal limb cellulitis. Equine gastric ulcer syndrome was reported in 3/7 (38%) as well. One horse also presented with anhidrosis.

The 4 horses that presented with a history of increased rectal temperature had the following co-morbidities which could have contributed to fever: bacterial cholangiohepatitis, cellulitis and anhidrosis, pleuropneumonia, and EMPF.

Euthanasia was elected due to progression of disease in all 8 cases. Complications that developed included hepatic encephalopathy (4), progressive inappetence and weight loss (4), and gastric impaction (1). Horse LA presented with concurrent EMPF and developed multi-organ failure with high fevers, severe weight loss, and severe bilateral renal hematuria. EMPF with EHV-5 infection was confirmed histologically, however the cause of renal hematuria was not evident on post-mortem examination.
