# Supplementary material for: Chronic hepatitis in horses with persistent equine hepacivirus infection
Source: Equine Vet J. 2025 Dec 25;58(2):444–57. doi: 10.1111/evj.70124 (PMC12892389; doi:10.1111/evj.70124)

Equine Veterinary Journal
BEVA

Figure S1. Examples of RNA integrity assessment in formalin-fixed paraffin embedded equine liver samples using in situ hybridisation of the equine PPIB housekeeping gene. Expression should be ubiquitous in equine hepatocytes. (A) Poor RNA quality with <10 puncta per cell (B) Fair RNA quality with 10-20 puncta per cell and (C) Good RNA quality with >21 puncta per cell, 400X, PPIB ISH. Samples with poor quality labelling with PPIB were not evaluated by EqHV ISH.

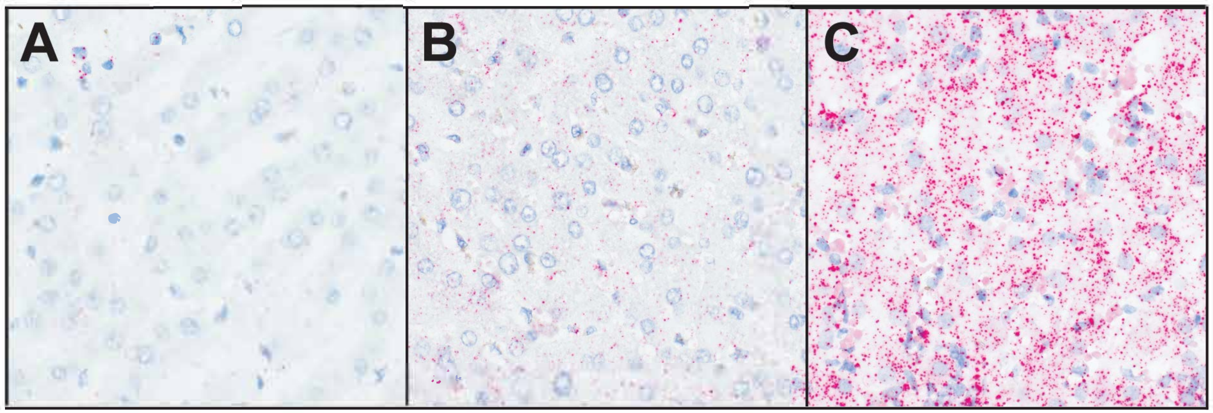

Supplement: Supplementary file 2 — Figure S1. Examples of RNA integrity assessment in formalin‐fixed paraffin embedded equine liver samples using in situ hybridisation of the equine PPIB housekeeping gene. Expression should be ubiquitous in equine hepatocytes. (A) Poor RNA quality with <10 puncta per cell (B) Fair RNA quality with 10–20 puncta per cell and (C) Good RNA quality with >21 puncta per cell, 400X, PPIB ISH. Samples with poor quality labelling with PPIB were not evaluated by EqHV ISH. [file EVJ-58-444-s007.pdf]
