# Supplementary figures and images for: Chronic hepatitis in horses with persistent equine hepacivirus infection
Source: Equine Vet J. 2025 Dec 25;58(2):444–57. doi: 10.1111/evj.70124 (PMC12892389; doi:10.1111/evj.70124)

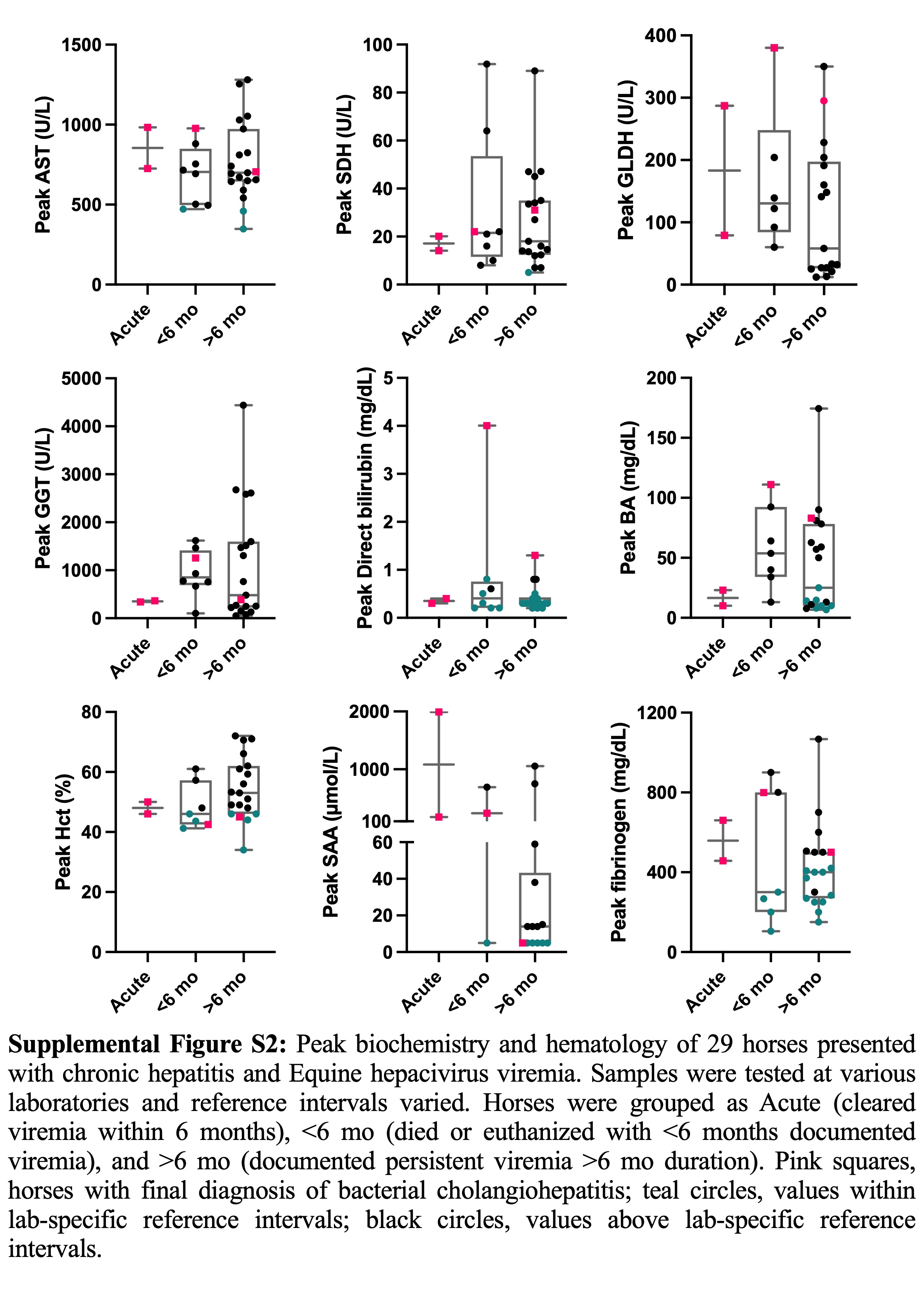

Supplement: Supplementary file 3 — Figure S2. Peak biochemistry and haematology of all 29 horses presented with chronic hepatitis and equine hepacivirus viremia. Samples were tested at various laboratories and reference intervals varied. Horses were grouped as Acute (cleared viremia within 6 months), <6 mo. (died or euthanised with <6 months documented viremia), and >6 mo. (documented persistent viremia >6 mo. duration, included in main manuscript). Pink squares, horses with final diagnosis of bacterial cholangiohepatitis; teal circles, values within lab‐specific reference intervals; black circles, values above lab‐specific reference intervals. [file EVJ-58-444-s002.jpg]

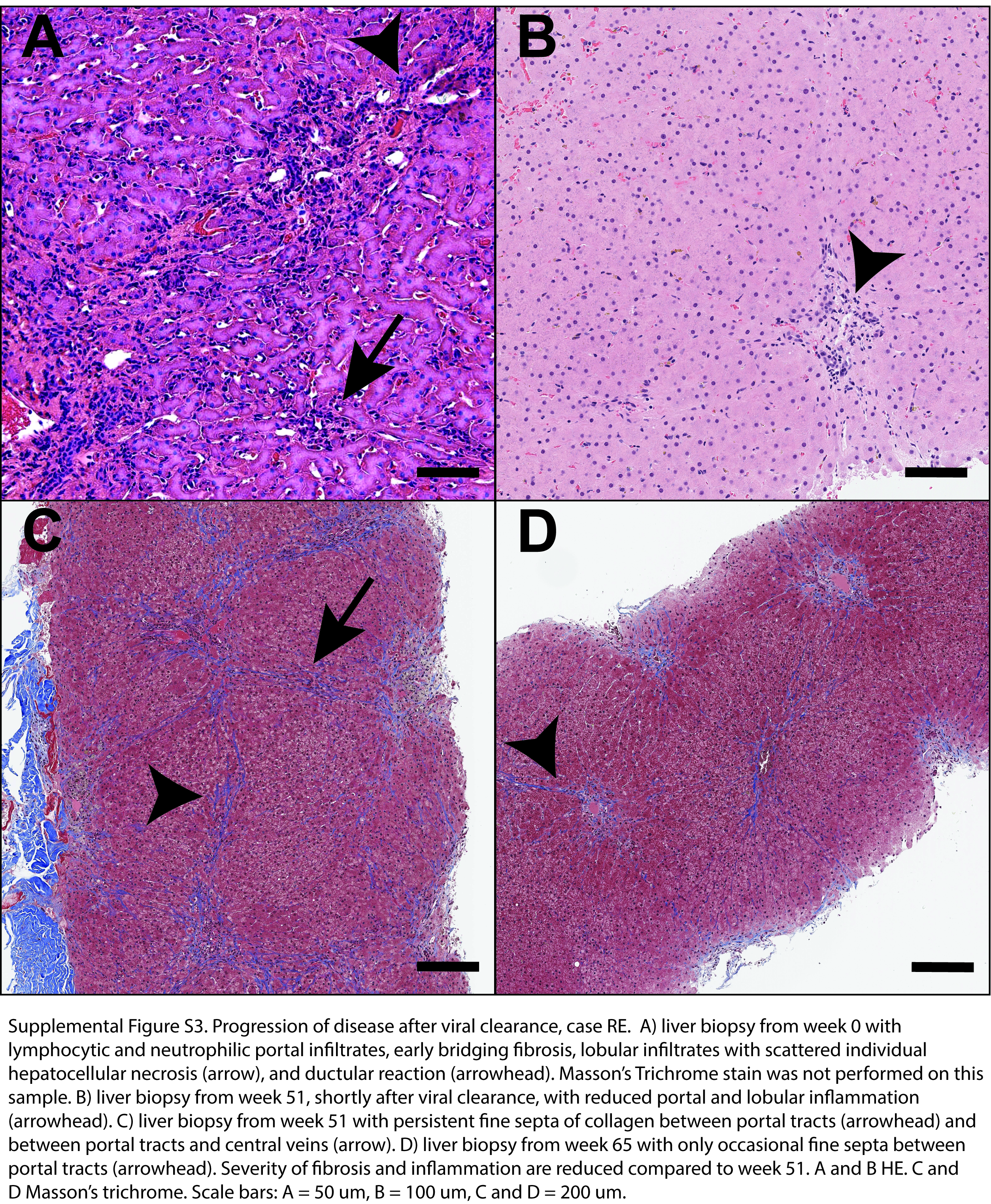

Supplement: Supplementary file 4 — Figure S3. Progression of disease after viral clearance, case RE. (A) Liver biopsy from week 0 with lymphocytic and neutrophilic portal infiltrates, early bridging fibrosis, lobular infiltrates with scattered individual hepatocellular necrosis (arrow), and ductular reaction (arrowhead). Masson's trichrome stain was not performed on this sample. (B) Liver biopsy from week 51, shortly after viral clearance, with reduced portal and lobular inflammation (arrowhead). (C) Liver biopsy from week 51 with persistent fine septa of collagen between portal tracts (arrowhead) and between portal tracts and central veins (arrow). (D) Liver biopsy from week 65 with only occasional fine septa between portal tracts (arrowhead). Severity of fibrosis and inflammation are reduced compared to week 51. A and B HE. C and D Masson's trichrome. Scale bars: A = 50 μm, B = 100 μm, C and D = 200 μm. [file EVJ-58-444-s008.tif]

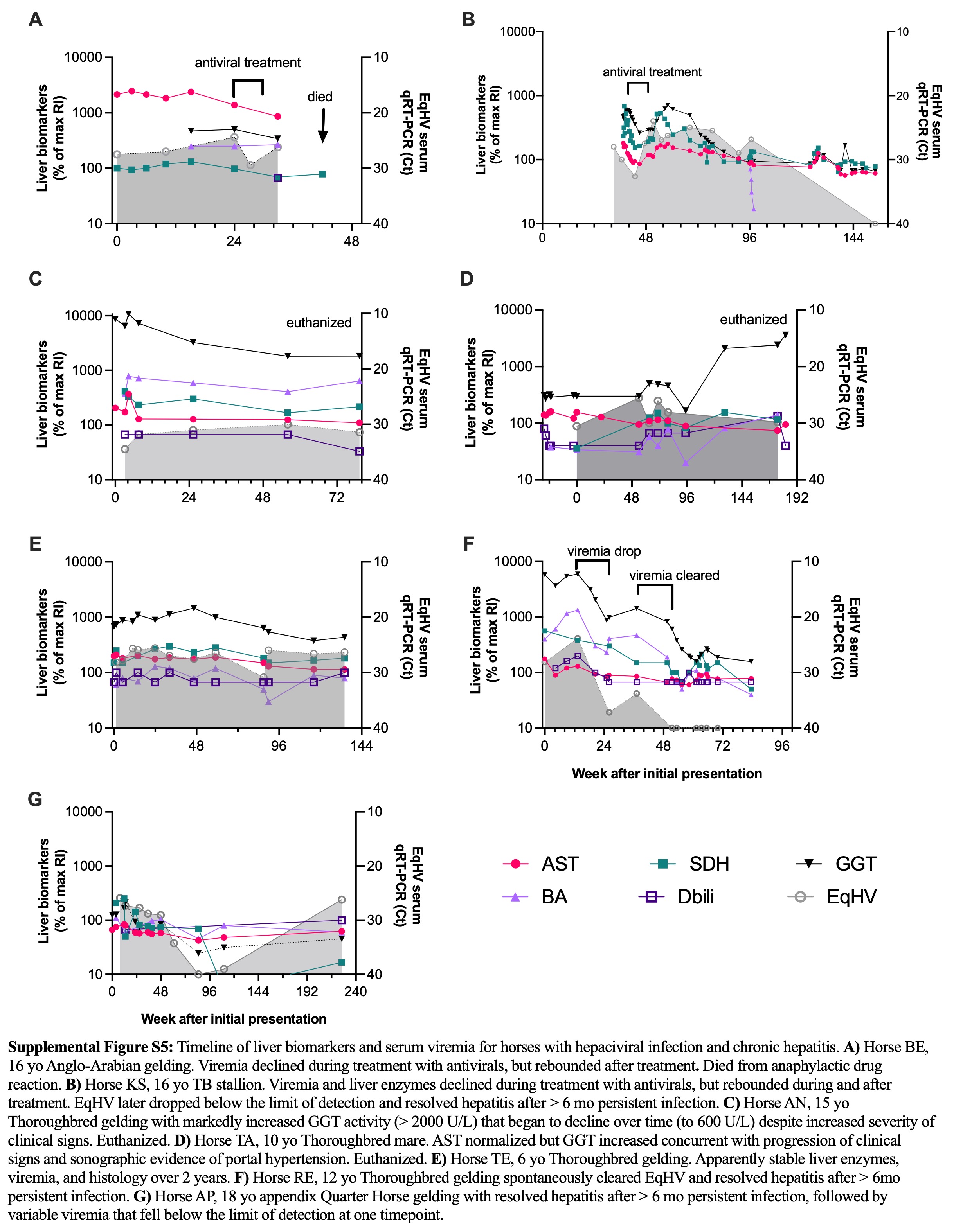

Supplement: Supplementary file 6 — Figure S5: Timeline of liver biomarkers and serum viremia for horses with hepaciviral infection and chronic hepatitis. (A) Horse BE, 16‐year‐old Anglo‐Arabian gelding. Viremia declined during treatment with antivirals, but rebounded after treatment. Died from anaphylactic drug reaction. (B) Horse KS, 16‐year‐old TB stallion. Viremia and liver enzymes declined during treatment with antivirals, but rebounded during and after treatment. EqHV later dropped below the limit of detection and resolved hepatitis after >6 mo persistent infection. (C) Horse AN, 15‐year‐old Thoroughbred gelding with markedly increased GGT activity (>2000 U/L) that began to decline over time (to 600 U/L) despite increased severity of clinical signs. Euthanised. (D) Horse TA, 10‐year‐old Thoroughbred mare. AST normalised but GGT increased concurrent with progression of clinical signs and sonographic evidence of portal hypertension. Euthanised. (E) Horse TE, 6‐year‐old Thoroughbred gelding. Apparently stable liver enzymes, viremia, and histology over 2 years. (F) Horse RE, 12‐year‐old Thoroughbred gelding spontaneously cleared EqHV and resolved hepatitis after >6 months persistent infection. (G) Horse AP, 18‐year‐old appendix Quarter Horse gelding with resolved hepatitis after >6 months persistent infection, followed by variable viremia that fell below the limit of detection at one timepoint. [file EVJ-58-444-s005.jpg]
