# Supplementary material for: Chronic hepatitis in horses with persistent equine hepacivirus infection
Source: Equine Vet J. 2025 Dec 25;58(2):444–57. doi: 10.1111/evj.70124 (PMC12892389; doi:10.1111/evj.70124)

Equine Veterinary Journal BEVA

Figure S4: Additional examples of histopathologic features of cases with documented persistent hepaci-virus infection. A) Dissecting fibrosis (arrow) separating and isolating islands of hepatocytes (arrowhead), case BE. B) Septal and dissecting sinusoidal fibrosis (arrow), case ZG. C) dense lymphoid aggregate (arrowhead) expanding a portal tract, case BE. D) clustered necrotic hepatocytes (arrow) and adjacent macrophages and lymphocytes (arrow-head), case BE. E) Collapse of the reticulin meshwork and condensation of fibres in sinusoids (arrowhead), case BE. F) Mild ductular reaction indicated by cords of progenitor cells that breach the limiting plate and transition into hepat-ic cords (arrowheads), case RE. A and B Masson's trichrome. C, D, and F HE. E reticulin. Scale bars: A-C, E= 100 um; A inset= 800 um; B inset= 200 um; D= 20 um; F= 50 um.

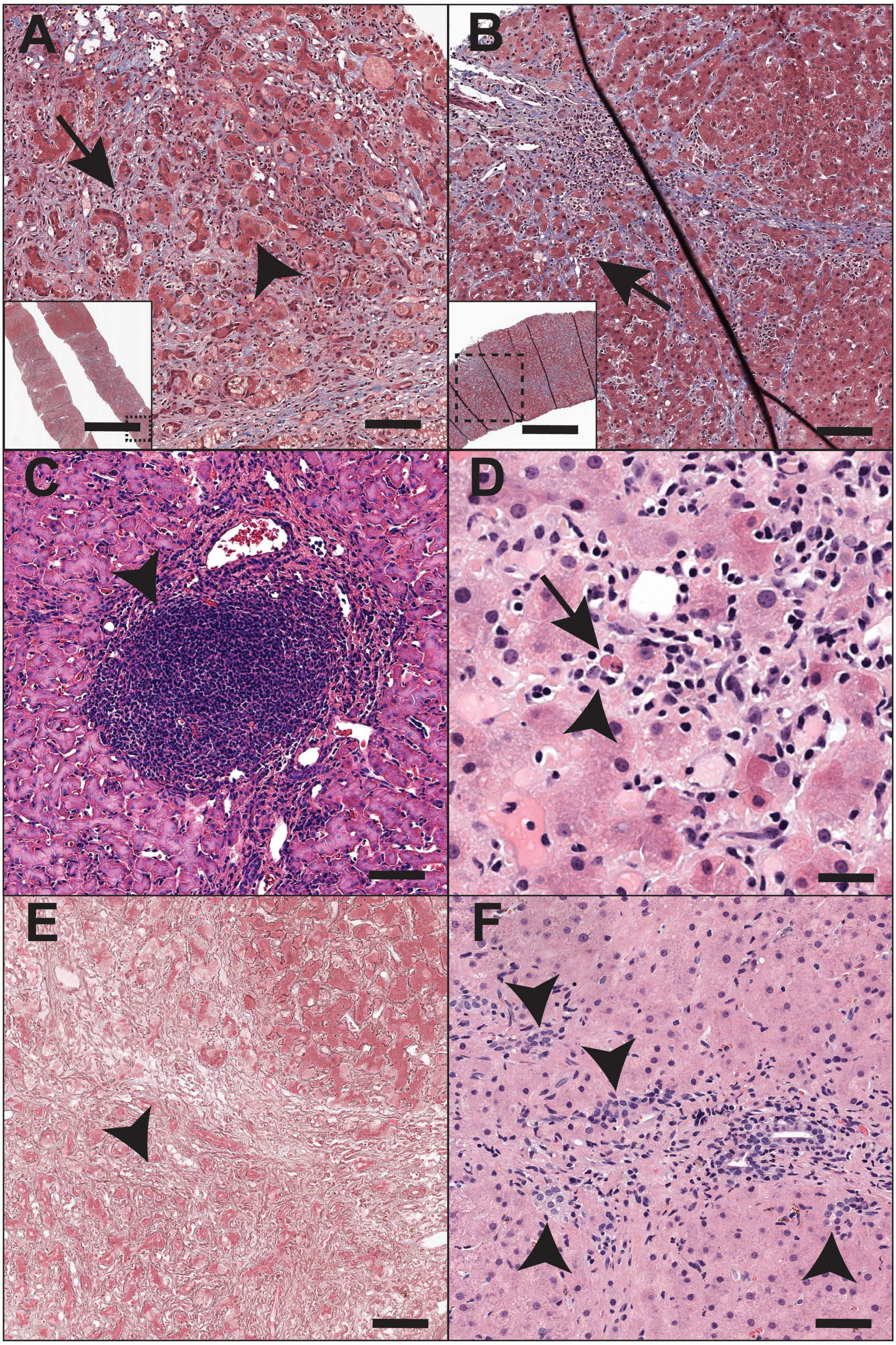

Supplement: Supplementary file 5 — Figure S4: Additional examples of histopathological features of cases with documented persistent hepacivirus infection. (A) Dissecting fibrosis (arrow) separating and isolating islands of hepatocytes (arrowhead), case BE. (B) Septal and dissecting sinusoidal fibrosis (arrow), case ZG. (C) Dense lymphoid aggregate (arrowhead) expanding a portal tract, case BE. (D) Clustered necrotic hepatocytes (arrow) and adjacent macrophages and lymphocytes (arrowhead), case BE. (E) Collapse of the reticulin meshwork and condensation of fibres in sinusoids (arrowhead), case BE. (F) Mild ductular reaction indicated by cords of progenitor cells that breach the limiting plate and transition into hepatic cords (arrowheads), case RE. A and B Masson's trichrome. C, D, and F HE. E reticulin. Scale bars: A–C, E = 100 μm; A inset = 800 μm; B inset = 200 μm; D = 20 μm; F = 50 μm. [file EVJ-58-444-s004.pdf]
