# Supplementary material for: Chronic hepatitis in horses with persistent equine hepacivirus infection
Source: Equine Vet J. 2025 Dec 25;58(2):444–57. doi: 10.1111/evj.70124 (PMC12892389; doi:10.1111/evj.70124)

Equine Veterinary Journal BEVA

Figure S6: Sonographic findings in horses with bacterial cholangiohepatitis and equine hepacivirus infection. A) Hyperechoic liver with biliary distention and multiple hyperechoic, variably shadowing foci consistent with choleliths in a horse with confirmed bacterial cholangiohepatitis. Horse QL. Left 7th intercostal space. B) Parallel channel sign indicating distended bile ducts. Horse OW. Right abdomen.

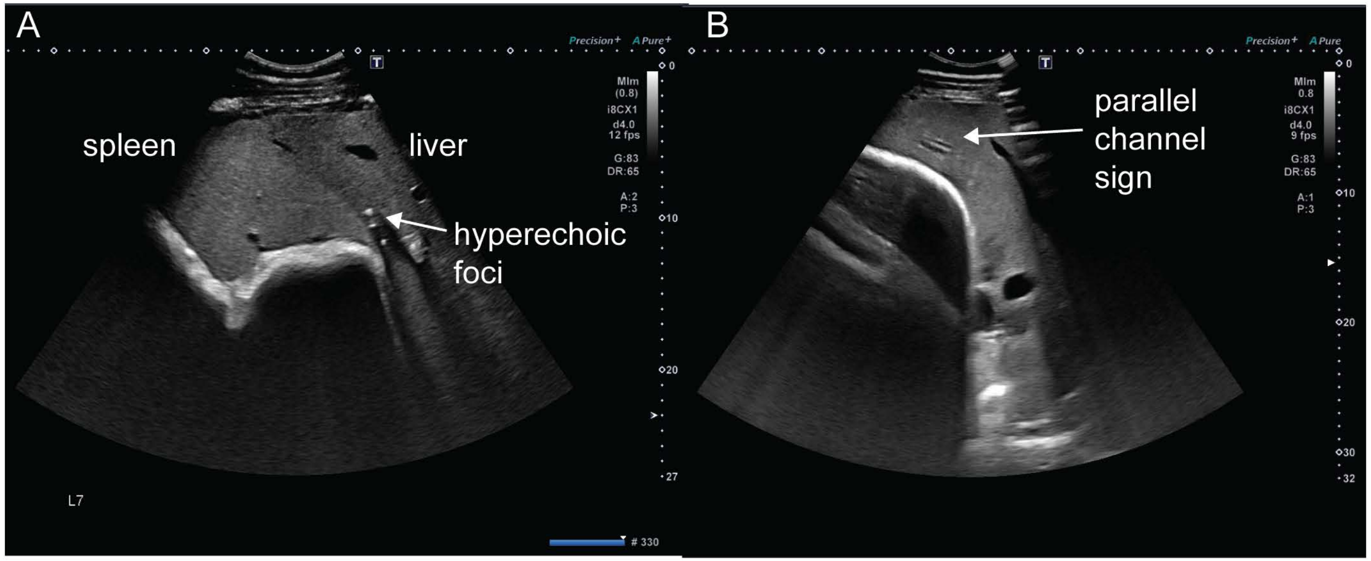

Supplement: Supplementary file 7 — Figure S6. Sonographic findings in horses with bacterial cholangiohepatitis and equine hepacivirus infection. (A) Hyperechoic liver with biliary distention and multiple hyperechoic, variably shadowing foci consistent with choleliths in a horse with confirmed bacterial cholangiohepatitis. Horse QL. Left 7th intercostal space. (B) Parallel channel sign indicating distended bile ducts. Horse OW. Right abdomen. [file EVJ-58-444-s009.pdf]
