# Supplementary material for: Chronic hepatitis in horses with persistent equine hepacivirus infection
Source: Equine Vet J. 2025 Dec 25;58(2):444–57. doi: 10.1111/evj.70124 (PMC12892389; doi:10.1111/evj.70124)

Equine Veterinary Journal BEVA

Figure S7: Histopathologic features of cases WE and OW. A) Severe portal to portal bridging fibrosis (asterisk), case WE. B) Severe portal-to-portal fibrosis (asterisk) with extension into sinusoids (arrow), case OW. C) Mild ductular reaction with progenitor cells extending into hepatic cords (arrowhead), case OW. D) Collapsed reticulin meshwork (asterisk) indicating parenchymal loss adjacent to normal reticulin (arrowhead), case WE. E) collapsed reticulin meshwork (asterisk) bridging between portal tracts and adjacent to normal reticulin (arrowhead), case OW. F) Peripheralisation of bile ducts with extension into hepatic cords (ductular reaction, arrowhead) and inflammatory cells (arrow), case OW. A and B Masson's trichrome. C and F HE. D and **E** reticulin. Scale bars: A, D inset, **E**= 200 um; B= 100 um; C, D, **F** inset= 50 um; A inset, **E** inset= 500 um; B inset, **F**= 800 um.

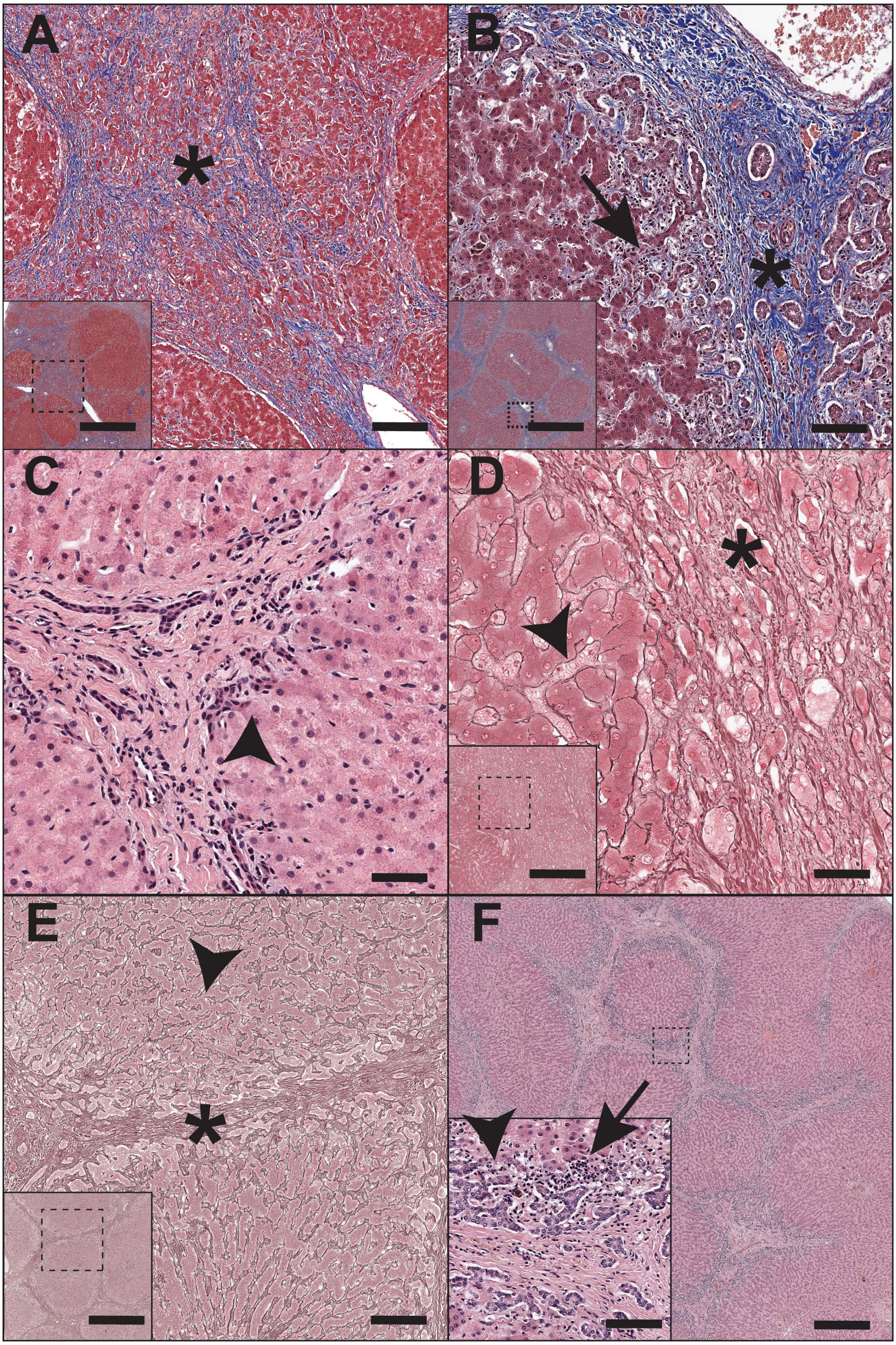

Supplement: Supplementary file 8 — Figure S7: Histopathologic features of cases WE and OW. (A) Severe portal to portal bridging fibrosis. [file EVJ-58-444-s010.pdf]
