# Supplementary material for: Chronic hepatitis in horses with persistent equine hepacivirus infection
Source: Equine Vet J. 2025 Dec 25;58(2):444–57. doi: 10.1111/evj.70124 (PMC12892389; doi:10.1111/evj.70124)
Supplement: Supplementary file 10 — Table S2. Peak biochemical markers and complete blood count findings for horses enrolled with chronic hepatitis and equine hepacivirus viremia. Horses were grouped as Acute (cleared viremia within 6 months), <6 mo. (died or euthanised with <6 months documented viremia), and >6 mo. (documented persistent viremia >6 mo. duration, included in main manuscript). Data presented as median (range). [file EVJ-58-444-s001.pdf]

Equine Veterinary Journal BEVA

Table S2: Peak biochemical markers and complete blood count findings for horses enrolled with chronic hepatitis and equine hepacivirus viremia. Horses were grouped as Acute (cleared viremia within 6 months), <6 mo (died or euthanised with <6 months documented viremia), and >6 mo (documented persistent viremia >6 mo duration). Horses in the >6 mo. group were additionally subset by presence or absence of equine parvovirus-hepatitis (EqPV-H co-infection). Data presented as median (range). Hct, haematocrit; Hgb, haemoglobin; RBC, red blood cell; PCV, packed cell volume; WBC, white blood cell; AST, aspartate aminotransferase; SDH, sorbitol dehydrogenase; GLDH, glutamate dehydrogenase; GGT, gamma glutamyl-transferase; BA, bile acids; Tbili, total bilirubin; Dbili, direct bilirubin; CK, creatine kinase; TG, triglycerides; Fe, iron; TIBC, total iron binding capacity; Fe Sat, iron saturation; SAA, serum amyloid A; TP, total protein.

	Acute (n=2)	Survived < 6 mo (n=8)	Persistent infection > 6 mo (n=19)	Persistent infection, EqPV-H neg (n=10)	Persistent infection, EqPV-H pos (n=9)
Hct (%)	46 - 50	46 (41.2 - 61)	53 (34 - 72)	51 (34 - 71)	53.3 (46 - 72)
Hgb (g/dL)	16.1 - 16.7	16.7 (14.8 - 21.9)	17 (11 - 25)	17.1 (11.4 - 24.4)	18.9 (15.7 - 25)
RBC (mill/µL)	8.8 - 9.4	9.98 (8.56 - 13.3)	10 (7 - 16)	9.3 (6.76 - 15.9)	11.4 (8.8 - 15.19)
PCV (%)	44 - 44	50.5 (39 - 61.4)	47 (37 - 66)	52.5 (43 - 66)	47 (37 - 66)
WBC (thou/µL)	9 - 11.12	10.98 (5.64 - 27.9)	10 (6.9 - 20)	9.2 (6.9 - 14.8)	11.4 (8.87 - 20.29)
AST (U/L)	726 - 890	705 (472 - 977)	699 (348 - 1280)	775.5 (542 - 1280)	655 (348 - 1254)
SDH (U/L)	14 - 20	22 (8 - 91.9)	18 (5 - 89)	24.5 (7 - 89)	14.6 (5 - 47.1)
GLDH (U/L)	79 - 287	131 (60 - 380)	58 (12 - 350)	144.5 (21 - 350)	32 (12 - 228)
GGT (U/L)	340 - 366	853 (102 - 1619)	483 (54 - 4441)	624.5 (54 - 4441)	269 (76 - 2585)
BA (µmol/L)	10 - 23	54 (13 - 111)	25 (6.8 - 174)	53.55 (9 - 174.4)	14.7 (6.8 - 81)
Tbili (mg/dL)	2.2 - 5.1	3.2 (1.3 - 9.5)	2.4 (1.3 - 6.9)	2.1 (1.3 - 6.9)	2.7 (1.7 - 4.7)
Dbili (mg/dL)	0.3 - 0.3	0.4 (0.2 - 4)	0.3 (0.2 - 1.3)	0.3 (0.2 - 1.3)	0.3 (0.2 - 0.5)
CK (U/L)	341 - 393	280 (164 - 789)	388 (196 - 14898)	412.5 (202 - 1030)	352 (196 - 14898)
TG (mg/dL)	53 - 65	65 (28 - 95)	53 (25 - 189)	64 (25 - 189)	51 (30 - 106)
Fe (µg/dL)	239 - 239	202 (168 - 235)	246 (52 - 344)	251 (178 - 316)	241 (52.3 - 344)
TIBC (µg/dL)	528 - 528	528 (478 - 578)	579 (413 - 761)	576 (499 - 761)	578.5 (413 - 829)
Fe Sat (%)	49 - 49	42 (35 - 48)	48.5 (33 - 66)	42.5 (36 - 66)	50 (33 - 63)
SAA (µg/mL)	174 - 1990	240 (5 - 690)	14 (5 - 1055)	9.5 (5 - 1055)	14 (5 - 749)
Fibrinogen (mg/dL)	457 - 660	300 (104 - 900)	400 (150 - 1067)	413.5 (150 - 600)	371 (200 - 1067)
TP (g/dL)	6.4 - 8.4	6.95 (5.6 - 8.6)	7.6 (6.2 - 9)	7.75 (6.6 - 8.5)	7.1 (6.2 - 9)
Globulins (g/dL)	3.1 - 3.1	2.8 (2.1 - 5.2)	4.2 (2.4 - 32)	4 (3 - 5)	4.2 (2.4 - 32)
Ammonia (µg/dL)	0 - 0	116 (46 - 186)	165 (3.9 - 409)	232 (55 - 409)	139.5 (3.9 - 275)
